# *CALB2* is a Mechanoresistance Gene in Metastatic Prostate Cancer

**DOI:** 10.1002/advs.76535

**Published:** 2026-07-20

**Authors:** Abigail R. Fabiano, Allen C. Luo, Paul Taufalele, Jenna A. Dombroski, Ehsan Aalaei, Schyler J. Rowland, Melissa S. Cantú, Alexandria T. Carter, Samantha V. Knoblauch, Cynthia A. Reinhart‐King, Michael R. King

**Affiliations:** ^1^ Department of Biomedical Engineering Vanderbilt University Nashville Tennessee USA; ^2^ Department of Bioengineering Rice University Houston Texas USA

**Keywords:** calretinin, fluid shear stress, mechanoresistant, mechanotransduction, metastasis

## Abstract

It remains unclear how circulating tumor cells (CTCs) adapt to and survive mechanical forces in the bloodstream, including fluid shear stress (FSS), making it critical to define the mechanisms that enable their survival and promote metastasis. To model these forces, we generated “mechanoresistant” (MR) LNCaP and PC3 prostate cancer (PCa) cell lines by repeatedly exposing cells to high‑intensity (HI) FSS (3950 dyn/cm^2^). LNCaP MR cells acquired marked resistance to HI FSS, exhibiting significantly reduced apoptosis, while PC3 cells demonstrated innate resistance. Bulk RNA‑sequencing revealed that LNCaP and PC3 MR cells developed distinct molecular programs. *CALB2* (calretinin) emerged as a mechanoresistance gene selectively upregulated in PC3 MR cells, and *CALB2* knockout significantly reduced viability upon re‑exposure to HI FSS. Analysis of *CALB2*‑high PCa cases captured patient‑level molecular heterogeneity, suggesting a mechanoadaptive state rather than a uniform aggressiveness‐driven tumor phenotype. This was consistent with the absence of correlation between *CALB2* levels and Gleason Scores. In an orthotopic PCa mouse model, the PC3 MR condition displayed the most aggressive early tumor growth and the largest endpoint tumor volumes, consistent with enhanced proliferation. Together, these findings demonstrate that PCa cells acquire discrete mechanoadaptive phenotypes under extreme FSS, revealing targetable pathways to limit metastatic competence.

## Graphical abstract

Prostate cancer cell lines of differing origins were cultured over many passages while being subjected to semi‐lethal levels of fluid shear stress, representing the harsh mechanical environment these cells are subjected to in the circulation during the process of distant metastasis. The resulting “mechanoresistant” prostate cancer cells showed interesting gene expression and phenotypic differences.

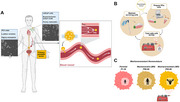

## 1 Introduction

In prostate cancer (PCa), the five‐year survival rate decreases by ∼70% when distant metastases develop [1, 2]. Cancer metastasis, or the spread of cancer, is a cascade of events that begins when tumor cells escape a primary tumor and invade the local tissue, followed by intravasation into the surrounding vascular or lymphatic system. Next, the cells must survive dissemination through the circulatory system. During this step, tumor cells are referred to as circulating tumor cells (CTCs). Thereafter, CTCs may extravasate out of the bloodstream to form secondary metastatic lesions. Despite the complexity of the metastatic cascade, only a small fraction of tumors cells (∼0.01%) survive circulation through the bloodstream, attributed to harsh physiological conditions the CTCs experience during circulation, essentially creating a situation where the cells with highest fitness are selected from the initial population. These harsh conditions may include adhesive forces between the vascular wall and CTCs, destruction by immune surveillance, and physiological fluid shear stress (FSS) [3, 4].

Androgens, such as testosterone, are the main initiators of PCa. First‐line treatments for PCa include androgen deprivation therapies (ADT) to reduce androgen levels and reduce tumor cell growth and proliferation [5, 6]. Despite this first line treatment option, often the cancer will progress to metastatic castration‐resistant prostate cancer (mCRPC). At this stage, the cancer cells stop responding to ADT, and CTCs no longer require androgens to survive (i.e. androgen‐independent), and acquire other means of metastasizing and remaining viable in the body. However, most research to elucidate the molecular characterization and phenotypes presented by CTCs surviving in the bloodstream are derived from liquid biopsies of PCa patients who present advanced stages of mCRPC. This makes it difficult to study and understand characteristic changes that CTCs undergo as the cancer proceeds to higher grades. Therefore, cancer cell lines that recapitulate the transition from localized to advanced disease are needed to better reveal the underlying mechanisms of metastasis.

Cells can sense and translate extracellular mechanical signals from physiological forces, into biochemical responses within the body through a process termed “mechanotransduction” [7, 8]. Within the circulatory system, low‐intensity (LI) FSS ranges from 0.50–4.0 dyn/cm^2^ in the venous compartment and from 4.0–30.0 dyn/cm^2^ within the arterial system [9, 10, 11]. CTCs can also experience brief, high‐intensity (HI) pulses of FSS on the order of 1000 dyn/cm^2^ at regions of turbulent flows, such as arterial bifurcations or within the heart [12, 13, 14, 15]. In most cancers, these mechanical cues influence metastatic potential and disease progression through both cancer‐promoting and cancer‐preventing mechanisms.

To model HI FSS in vitro, a syringe pump is conventionally used, in which cells are perfused through a small bore needle to supply an average FSS >1000 dyn/cm^2^ and produce a brief pulse of elevated shear [8, 9, 16]. Syringe pump–based systems have been commonly used to model high‐intensity FSS because they enable precise control over shear magnitude and exposure duration, while maintaining high reproducibility. In contrast, these parameters are difficult to independently control in turbulent flow systems. Few studies exist that help delineate why only a small fraction of CTCs can survive exposure to harsh forces, particularly FSS in the bloodstream. Most of these studies investigate the effects of FSS on the viability of cancer cells, as well the effects on various factors such as actin cytoskeletal remodeling, membrane damage and repair, and partial or full epithelial‐to‐mesenchymal transitions (EMT) [9, 17]. Most studies have shown that increased FSS intensity reduces cell proliferation, generally consistent with reduced viability [12, 18]. Evidence shows that non‐transformed cells are more vulnerable to cell death via exposure to HI FSS compared to cancer cells [9, 17]. Studies have shown that exposing CTCs to a single pulse of HI FSS induced resistance to subsequent pulses [9, 19, 20]. Our lab has also found that exposure of PCa and colorectal cancer cells to HI FSS resulted in higher apoptosis as the number of shear pulses increased. Cell lines of lower metastatic potential experienced more significant cell death at ten shear pulses compared to highly aggressive cell lines [11, 21]. Our group has also shown that nuclear protein lamin A/C is necessary to promote CTC survival under HI FSS [22]. Collectively, these previous HI FSS studies have not exceeded exposure to 3950 dyn/cm^2^.

Additionally, proteomic analyses has shown that distinct molecular programs may underlie the mechanoadaptation of CTCs in the bloodstream [9, 23]. Because of sparsity and limited access to patient‐derived CTCs, prior research has yet to report distinct molecular signatures that can reveal which tumor site an individual CTC is derived from, a critical step toward developing more targeted cancer therapies. A potential mechanoadaptive gene we chose to explore in this study is *CALB2*, identified from RNA‐sequencing data presented in the current work. *CALB2* encodes for the calretinin protein and is responsible for buffering intracellular calcium (Ca^2+^) levels, thus owing potential to mechanosensory signaling downstream of mechanosensitive ion channel (MSC) activation in cancer cells. Overexpression of calretinin in malignant mesothelioma cells increased the capacity of migration and invasion, and it was observed that calretinin co‐localizes with focal adhesion kinase (FAK) at focal adhesion sites [24]. The direct link between *CALB2*, mechanotransduction, and downstream signaling in cancer cells represents a critical knowledge gap in our understanding of metastatic cancer. Investigating the expression of *CALB2* in response to HI FSS exposure and its co‐expression levels with other mechanoadaptive genes could therefore provide insight toward conserved molecular signatures that facilitate metastatic competence.

The goal of this study was to raise and characterize PCa cell lines that are resistant to hemodynamic levels of FSS, termed “Mechanoresistant (MR),” to understand the adaptive response of CTCs traveling through the bloodstream during metastasis. This allows one to elucidate new, promising therapeutic targets. This is critical since CTC survival in circulation is the least studied step in metastasis due to the dynamic nature of this biological process, and it is unknown how long CTCs remain in the circulation, although it is speculated to be 25–30 min for single cells [25, 26]. Our approach consisted of raising MR cells in vitro through repeated exposure of PCa cells to increasing numbers of semi‐lethal pulses of HI FSS (3950 dyn/cm^2^), and to characterize the cells using in vitro and in vivo assays, with the goal of increasing the number of pulses the cells were exposed to with each iteration. We hypothesized that MR cells will exhibit different phenotypes and acquire changes in gene expression after ∼50 HI FSS treatments. We used HI FSS for these studies rather than LI FSS because there is a lack in studies that capture the survival mechanisms of CTCs under HI FSS. Additionally, based on previous research, HI FSS is much more likely to eliminate populations not resistant to FSS owing to the greater magnitude of exposure, allowing us to retain a pure population of cells that are FSS‐resistant in a more feasible manner [9, 11, 21].

There are currently no existing studies that have examined the repeated exposure of tumor cells to HI FSS over the period of days, weeks, or months. Previous results show variability between studies and exposure of cells to FSS has not exceeded 3950 dyn/cm^2^. Development and characterization of MR cell lines will yield a greater understanding of the mechanisms of CTC mechanoadaptation in the bloodstream, enabling the identification of more effective therapeutic strategies and molecular targets to treat patients with metastatic cancer.

## 2 Results

### 2.1 The Development of Mechanoresistant Cancer Cell Lines

**FIGURE 1:**
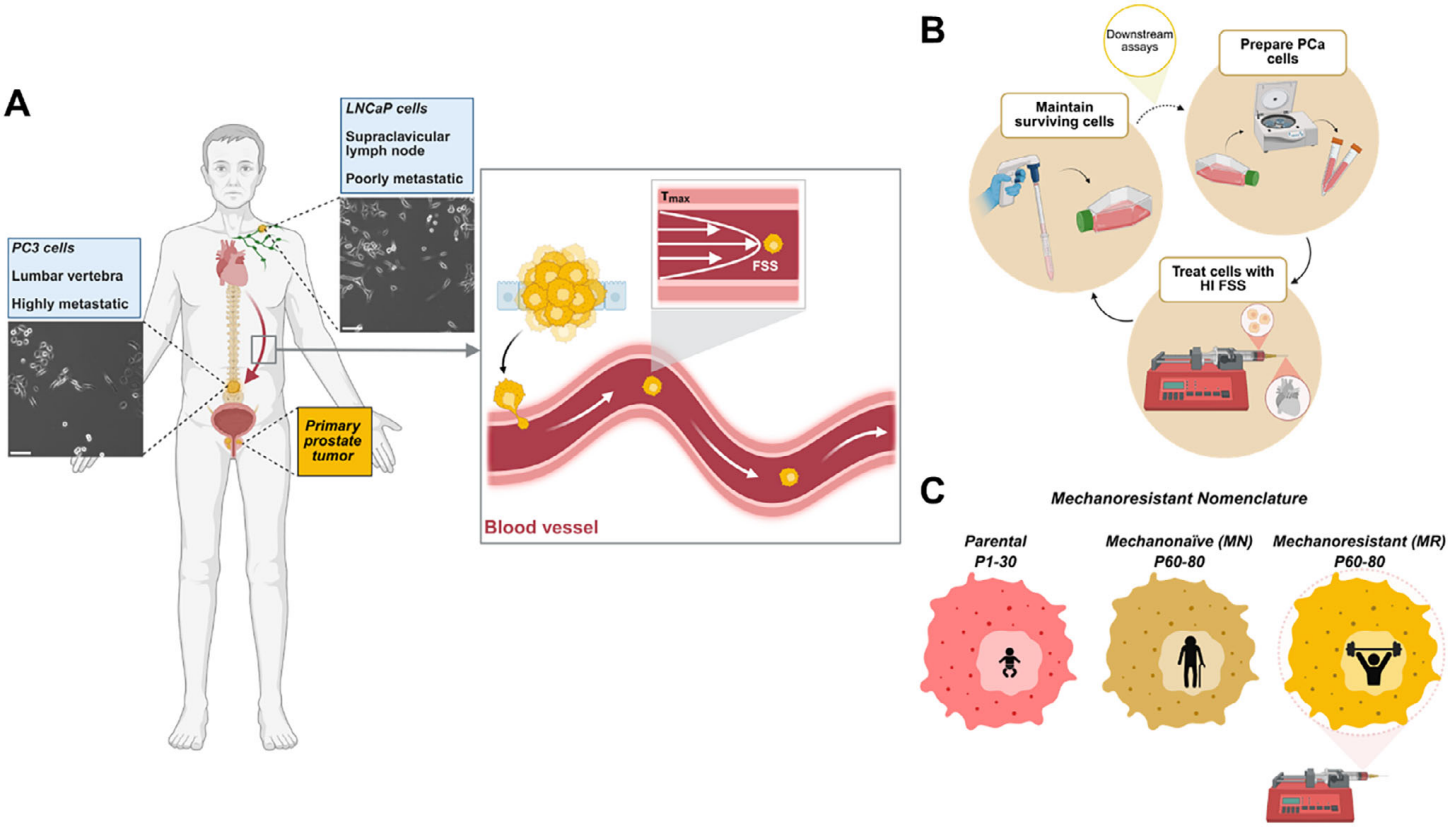
Experimental overview. (A) Prostate cancer (PCa) cell lines and anatomical origins. (B) Process overview for the generation of the mechanoresistant (MR) PCa cells. (C) Nomenclature for the parental, mechanonaïve and MR cells used in this study.

To differentiate the mechanoadaptive mechanisms of cells that govern different levels of metastatic potential, the development and characterization of the MR cells were completed on two different PCa cell lines (Figure 1A). LNCaP cells were originally derived from a lymph node metastasis, and likely did not experience circulatory levels of FSS >12 dyn/cm^2^ while migrating from the primary tumor, through soft tissue, to the lymph node [27, 28, 29]. PC3 cells were originally derived from bone metastases in the lumbar vertebrae, and therefore have experienced circulatory FSS in the body, which is known to reach values of at least 30–60 dyn/cm^2^ [29, 30]. Thus, we expect PC3 cells to be more innately resistant to FSS or harsh mechanical forces in comparison to LNCaP cells, all other factors being equal.

The development of MR cells is analogous to raising chemoresistant cancer cells (Figure 1B), where a pure population of cells is subjected to a semi‐lethal dose of drug, and cells resistant to chemotherapy are selected over time. In essence, this selects for the subpopulation of cells with the greatest fitness, and the resistant cells are found to retain this capacity for survival in subsequent treatments [31]. We raised the MR cells via repeated exposure to HI FSS using our established syringe pump method, in which cells are infused through a 30 G needle at 14 mL/min to produce a wall FSS within the needle of 3950 dyn/cm^2^ [9, 11]. It is estimated that the transit time is 1.08 ms for a cell to experience a single pulse through needle [13]. Following treatment, the cells were re‐plated and maintained in the incubator to recover. Between treatments, the MR cells were used for downstream assays to characterize and assess changes in behavior. Once the MR cells were recovered, they were treated with HI FSS, through increasing shear pulses over time, with the goal of selecting the fittest cells. Ultimately, we increased the number of HI FSS shear pulses that the cells were exposed to in each treatment cycle, reaching a 10‐shear pulse exposure maximum for the LNCaP cells and a 20‐shear pulse maximum for the PC3 cells due to differences in their innate FSS resistance, consistent with their physiological origin [27, 28, 29, 30]. Each shear pulse was administered two min apart with a calculated mean transit time of 1.08 ms [32]. Direct measurements of individual cell transit time through cardiac valves are not available. Considering the mm‐scale geometry of valve structures and measured velocities, it would be estimated that cells traverse these regions on millisecond timescales [33]. Although our procedure likely administers the lower end of estimated transit times, prior work has demonstrated that brief shear stress exposures of this duration are sufficient to elicit cellular mechanotransduction responses [9, 11, 21].

For the experiments outlined here, three conditions were tested for each cell line (Figure 1C). Two control conditions; including the unmodified parental cells at low passage (P1–P30), and the mechanonaïve (MN) condition, which represent unmodified parental cells at a higher passage (P60–P80) to control for any long‐term effects of cell culture that the MR cells may experience. The MR cells were used for characterization experiments when they reached ∼50–60 FSS treatments. When the MR cells reached ∼60 FSS treatments and the MN cells reached passage 60, the cell lines were authenticated using ATCC short tandem repeat (STR) profiling to confirm similar, recognizable profiles to the parental cells (Supplementary File 1) [34]. For the experiments outlined here, we examined the response of the MR cells at 4 and 24 h post‐HI FSS exposure. Additionally, the “MR” condition in figure panels refers to the MR cells immediately after they are taken from culture after recovering from the previous HI FSS treatment.

### 2.2 Mechanoresistant LNCaP Cells Demonstrate Significantly Enhanced Viability Following Fluid Shear Stress Exposure

**FIGURE 2:**
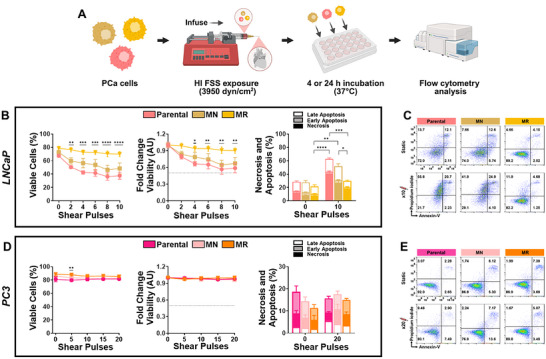
Viability following high‐intensity fluid shear stress exposure. (A) Procedural overview. (B) Percentage of viable LNCaP cells, fold change in viability, and total necrosis and apoptosis. (C) Representative LNCaP AV/PI flow cytometry plots. (D) Percentage of viable PC3 cells, fold change in viability, and total necrosis and apoptosis. (E) Representative PC3 AV/PI flow cytometry plots. The static conditions in (C, E) represent 0 shear pulses. *n* = 3 independent experiments. (B, D) Two‐way ANOVA. *p < 0.05, **p < 0.01, ***p < 0.005, ****p < 0.0001. Error bars represent mean ± SEM.

We used the annexin‐V/propidium iodide (AV/PI) flow cytometry assay to detect differences in the viability and apoptosis of the MR cells 4 and 24 h following HI FSS exposure (Figure 2A). For the LNCaP cells we did not exceed 10 shear pulses since these cells demonstrate lower innate resistance to FSS. Early experiments indicated that the parental and MN cells were ablated beyond 10 shear pulses, and additional pulses were not informative. Since the PC3 cells are more innately resistant to FSS, we tested up to 20 shear pulses per treatment of these cells [9, 11, 35]. Thus, the LNCaP cells were analyzed at 4 h and PC3 cells analyzed at 24 h post‐treatment. The conditions associated with 0 shear pulses represent static conditions, in which the samples were not exposed to any FSS. At 4 h, the LNCaP MR cells show significantly less apoptosis compared to the parental and MN cells (Figure 2B,C). At 10 shear pulses, the MR cells maintained 69.85 ± 3.76% (mean ± SEM) viability, while the parental and MN conditions reduced to 37.52 ± 5.23% and 48.40 ± 8.22%, respectively (Figure 2B). Most of the cell death occurred via necrosis at all conditions, with the total apoptosis and necrosis the lowest for the LNCaP MR cells overall at 10 shear pulses, and insignificant variations under static conditions. 24 h post‐FSS exposure, the LNCaP MR cells maintained higher viability at 10 shear pulses compared to the parental and MN conditions (Figure S1A,B). Overall, the PC3 parental, MN, and MR conditions all maintained nearly unchanged viability in response to HI FSS up to 20 shear pulses (Figure 2D,E). The total cell death remained below 20% for all conditions (Figure 2D). Similar trends were seen at 4 h post‐shear (Figure S1C,D). These results confirm that the PC3 cells are innately resistant to 3950 dyn/cm^2^ of FSS. However, determining the characteristics that distinguish PC3 MR from the parental and MN cancer cells lacking HI FSS exposure is imperative to understand the physiological adaptations driving elevated metastatic burden seen in patients.

### 2.3 Morphological Phenotypic Shifts are Observed in Mechanoresistant PC3 Cells

**FIGURE 3:**
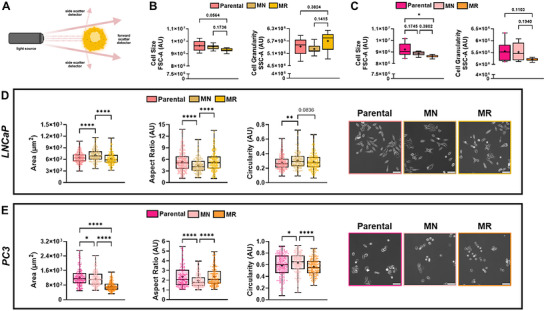
Mechanoresistant PC3 cells acquire distinct morphological features. (A) Schematic showing the forward scatter (FSC) and side scatter (SSC) generated by the flow cell in the flow cytometer (66 µL/min). (B) LNCaP and (C) PC3 cell FSC and SSC. (D) LNCaP cell morphology for adhered cells; area, aspect ratio, circularity and representative phase contrast images. (E) PC3 cell morphology for adhered cells; area, aspect ratio, circularity and representative phase contrast images. Scale bar = 100 µm. (B, C) *n* = 4–5 independent experiments. (D, E) *n* = 186–286 cells per condition. (B–E) One‐way ANOVA. (B, C) Box plots showing the 10–90th percentile with individual points shown as outliers, (+) indicates the mean. (D, E) Box plots showing all points, extending from the min to max, (+) indicates the mean. *p < 0.05, **p < 0.01, ***p < 0.005, ****p < 0.0001. Error bars represent mean ± SEM.

Morphological features of LNCaP and PC3 cells compared to their respective parental and MN conditions were examined under two different adherence states. Suspended cells were analyzed by flow cytometry (Figure 3A) to evaluate size via forward scatter (FSC) and granularity via side scatter (SSC) (Figure 3B,C). For adhered cells, phase‐contrast images were acquired and quantified using FIJI (Figure 3D,E) [36].

Overall, the LNCaP MR cells showed no significant changes in morphology compared to the corresponding parental cells for the suspension (Figure 3B) or adhered (Figure 3D) measurements. Interestingly, the LNCaP MN cells showed the most variation in morphology, exhibiting phenotypic drift, resulting from long‐term cell culture, as characteristics such as growth rate and cytoskeletal behavior can be affected [37, 38]. However, both the LNCaP and PC3 MR cells showed a 1.04‐ and 1.07‐fold decrease in cell size (FSC), respectively, compared to the parental cells (Figure 3B). The PC3 MR cells exhibited a 1.07‐fold decrease in granularity compared to the parental cells (Figure 3C). Consistent with a significantly decreased cell size, the PC3 MR cells also displayed a significantly lower cell area compared to the parental and MN conditions when adhered (Figure 3E). The aspect ratio is similar between the PC3 parental and MR cells, with a slight decrease in circularity observed for the PC3 MR cells (0.563 ± 0.0094) (Figure 3E). Collectively, the reduced cell size, spreading and granularity suggests that the PC3 MR cells may have adopted an amoeboid morphology [39, 40].

We measured vimentin and E‐cadherin protein expression using the Western blot assay to determine if there were any correlations between EMT status and morphological features. Typically, mesenchymal‐like cells show increased vimentin and decreased E‐cadherin expression. The LNCaP MR cells demonstrated a decrease in E‐cadherin expression compared to the parental and MN cells, however, vimentin expression remained undetectable at all three conditions (Figure S2A), showing no trend between morphology and EMT. The PC3 MR cells showed a significantly lower EMT ratio compared to the PC3 parentals (Figure S2B,C), measured by dividing the normalized vimentin expression by the normalized E‐cadherin expression. The PC3 MN condition shows a complete MET transition, with significantly higher amounts of E‐cadherin and significantly lower vimentin expression compared to the PC3 parental cells, likely showing effects from long‐term cell culture. The PC3 MR cells show an intermediate or hybrid EMT phenotype, with detectable levels of both vimentin and E‐Cadherin. For the PC3 MR cells, these observations may correlate with a potential amoeboid phenotype as the morphology data suggest [40, 41]. These results are consistent with recent studies that have shown that many CTCs undergo partial mesenchymal‐to‐epithelial transition (MET) while extravasating, supporting the idea that an intermediate phenotype promotes plasticity and progression through the metastatic cascade [42, 43, 44].

### 2.4 High‐Intensity Fluid Shear Stress Increases S Phase Activity in the Cell Cycle

**FIGURE 4:**
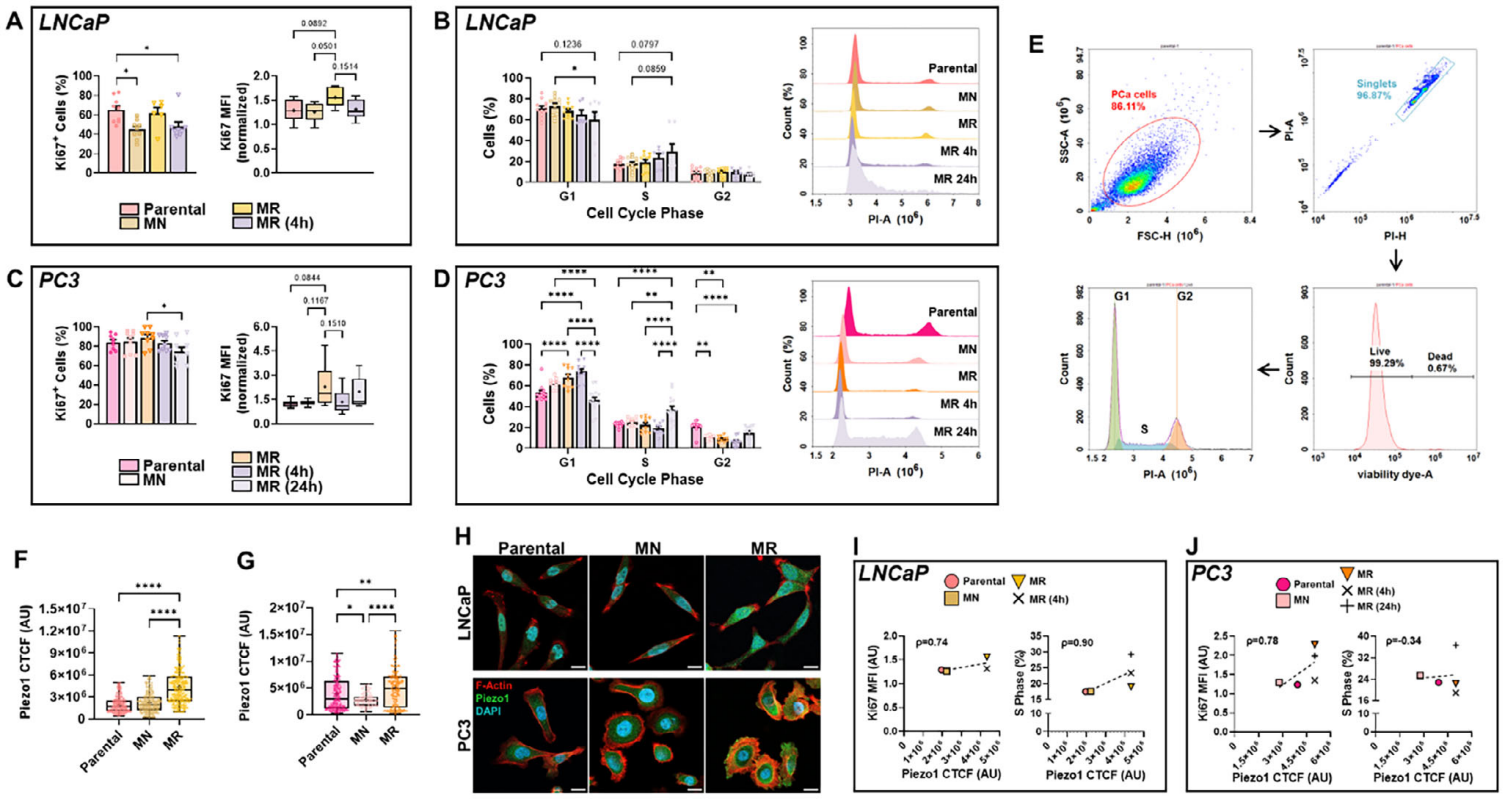
Mechanoresistant cells post‐shear show enhanced proliferative activity. (A) Percentage of proliferating cells and Ki67 median fluorescence intensity (MFI) for LNCaP cells. (B) Cell cycle analysis and representative histograms for LNCaP cells. (C) Percentage of proliferating cells and Ki67 MFI for PC3 cells. (D) Cell cycle analysis and representative histograms for PC3 cells. (E) Cell cycle flow cytometry gating scheme. Piezo1 corrected total cell fluorescence (CTCF) for (F) LNCaP and (G) PC3 cells measured using Equation 4. (H) Representative Piezo1 confocal images. Piezo1, Ki67, and S phase cell cycle correlation analysis for (I) LNCaP and (J) PC3 cells. (A‐D,I,J) *n* = 3–5 independent experiments. (F, G) *n* = 124–271 cells per condition. (A,C,F,G) One‐way ANOVA. (B,D) Two‐way ANOVA. (I,J) Spearman's two‐tailed correlation test. (A, C,) Box plots showing the 10–90th percentile, (+) indicates the mean. (F, G) Box plots showing all points, extending from the min to max, (+) indicates the mean. *p < 0.05, **p < 0.01, ***p < 0.005, ****p < 0.0001. Error bars represent mean ± SEM.

We measured proliferation using Ki67 analysis via flow cytometry (Figure 4A,C). Ki67 expression is detected when cells are in the G_1_, S or G_2_ phase of the cell cycle, including mitosis [45]. Typically, PC3 cells have been shown to exhibit higher proliferation compared to LNCaP cells due to their increased aggressiveness and faster doubling time, consistent with the trends observed when measuring the percentage of Ki67^+^ cells in these samples (Figure 4A,C) [46]. Overall, significant shifts in the percentage of Ki67^+^ cells were not observed. However, when examining the median fluorescence intensity (MFI) of Ki67 expression normalized to the isotype controls, the LNCaP and PC3 MR cells showed the highest intensity. Figure 4A shows that for the LNCaP MR cells, this value reached 1.561 ± 0.082, and 4 h post‐shear was 1.316 ± 0.071. The LNCaP parental and MN cells showed a mean value of 1.292 ± 0.074 and 1.262 ± 0.067, respectively. The Ki67 MFI for the PC3 MR cells was 2.284 ± 0.47, while the PC3 parental condition remained at 1.237 ± 0.073 (Figure 4C).

Since the Ki67 MFI was highest for the MR conditions in both cell lines, we chose to run a cell cycle analysis using flow cytometry to assess whether the elevated proliferative index corresponds to shifts in cell cycle distribution (Figure 4B,D). For this assay, the cells are stained with PI + RNase to allow the PI to intercalate to the DNA within the fixed cells, providing a histogram readout of PI intensity to gate for G_1_, S or G_2_ phase [47]. For the LNCaP cells, we decided to measure up to a 24 h timepoint post‐shear to evaluate if differences in cell cycle show similar trends to the PC3 MR cells for a more informative comparison. More significant variations in cell cycle were observed amongst the PC3 cell conditions, in which the MR cells 24 h after HI FSS exposure demonstrate a significant increase in the percentage of cells in S phase (36.70 ± 3.54%), consistent with a decreased cell percentage in G_1_ phase (46.03 ± 2.911%) relative to the other conditions (Figure 4D). The LNCaP MR cells 24 h post‐shear show a similar trend (Figure 4B). This suggests that the MR cells move out of G_1_ phase more readily. Additionally, the MR cells are not found in cell cycle arrest in the G_2_/M phase following FSS exposure, as seen previously with other cancer studies [48].

Piezo1 is a Ca^2+^‐gated MSC, and increased expression of Piezo1 in PCa tissues and cells (relative to benign conditions) has been observed [49, 50]. We measured Piezo1 expression in parental, MN and MR conditions for both cell lines via immunofluorescence (IF) confocal imaging. The LNCaP and PC3 MR cells showed significantly higher Piezo1 expression than in control cells (Figure 4F–H). Piezo1 expression was reduced in PC3 MN cells relative to the PC3 parental condition, likely due to phenotypic drift associated with extended in vitro culture [37, 38]. Piezo1 expression in the MR cells were measured ∼48 h following FSS exposure, as cells were allowed to grow on culture‐treated slides for two days following HI FSS treatment prior to fixation and permeabilization for confocal imaging. We compared the average Piezo1 expression with the average Ki67 MFI and S phase percentage for each condition to identify any correlations. For the MR cells at 4 and 24 h post‐treatment, we used the same average Piezo1 expression measured in Figure 4F,G as a baseline comparison. We observed that the Piezo1 expression positively correlates with the percentage of LNCaP cells in the S phase (Figure 4I). In PC3 cells, increased Piezo1 expression showed a positive correlation with increased proliferative index (Figure 4J).

### 2.5 RNA‐Sequencing Reveals Distinct Molecular Phenotypes Acquired by the Mechanoresistant Cells

**FIGURE 5:**
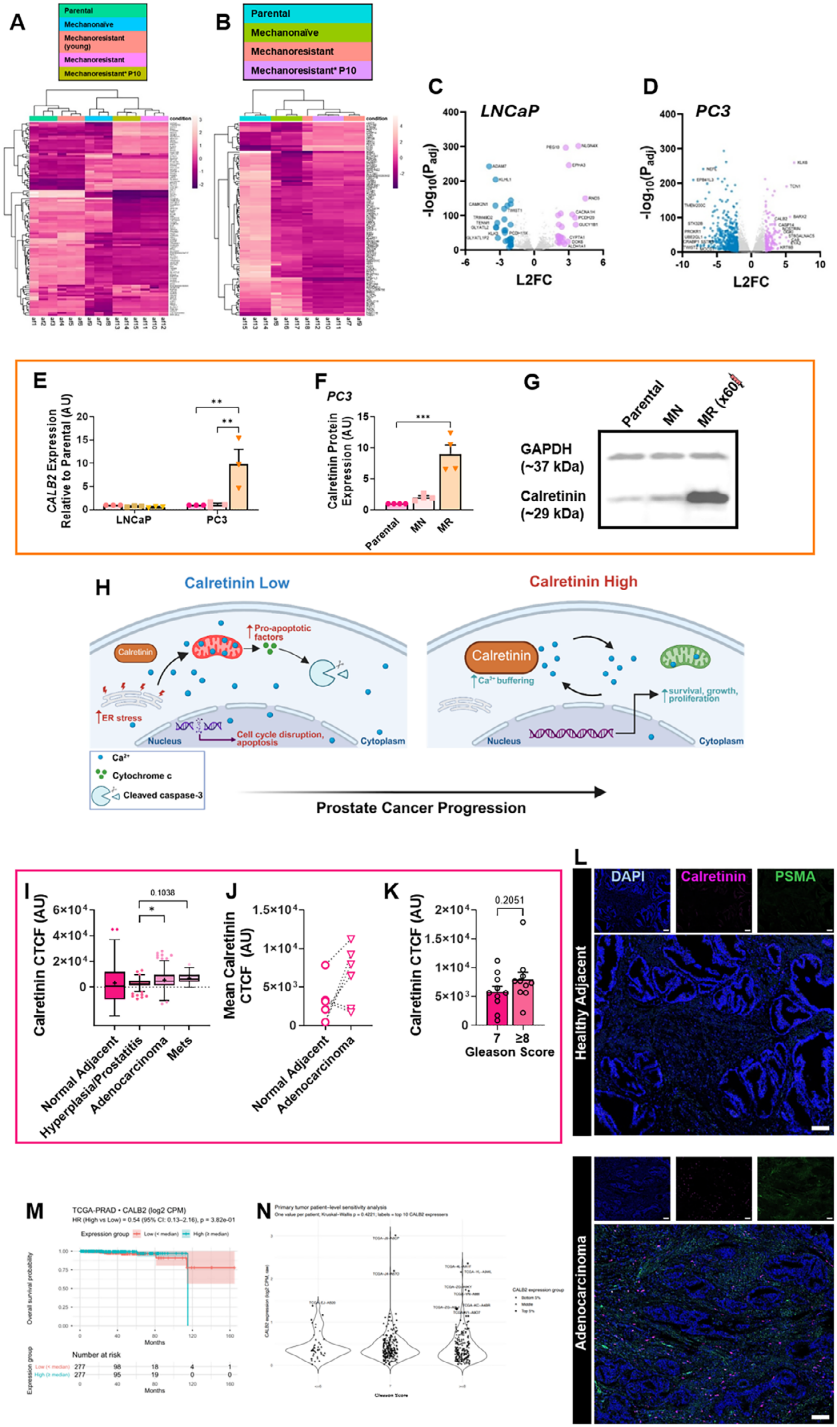
Distinct molecular phenotypes revealed within the mechanoresistant cells. Heatmap plots showing the top 100 differentially expressed genes for the (A) LNCaP and (B) PC3 cells. Volcano plot showing the significantly up‐ and down‐regulated genes in the (C) LNCaP mechanoresistant (MR) cells compared to the parental LNCaP cells and (D) PC3 MR cells compared to the PC3 parental cells. (E) CALB2 expression measured via qPCR normalized to GAPDH. (F) Calretinin protein expression measured using a Western blot assay, normalized to GAPDH. (G) Representative PC3 Western blots. (H) Schematic showing the function of calretinin in cancer cells at low and high expression. (I) Calretinin corrected total cell fluorescence (CTCF) expression in human tissue samples. (J) Mean calretinin CTCF in healthy adjacent tissue samples relative to their respective adenocarcinoma samples. (K) Calretinin CTCF in prostate cancer adenocarcinoma tissue samples grouped by Gleason Score. (L) Representative 20X images of calretinin and PSMA staining in human tissue samples, scale bar = 100 µM. (M) TCGA overall survival of prostate cancer patients with low and high CALB2 expression. (N) Violin plots of CALB2 expression correlated with Gleason Score from the TCGA‐PRAD cohort. CALB2‐high (top) and CALB2‐low (bottom) outliers were defined as the top and bottom 5% of CALB2 expression within the data set. (E, F) *n* = 3–5 independent experiments. (A–D) *n* = 3 RNA samples per condition. (I) *n* = 20–210 data points per condition. (J) *n* = 6 patients. (K) *n* = 10–11 adenocarcinoma tissue samples per Gleason Score. (E) Two‐way ANOVA. (F, I) One‐way ANOVA. (K) Welch's unpaired *t*‐test. (I) Tukey box plot, with outliers shown as individual points, (+) indicates the mean. (N) Krus–Wallis test. *p < 0.05, **p < 0.01, ***p < 0.005, ****p < 0.0001. Error bars represent mean ± SEM.

We performed bulk RNA‐sequencing for the LNCaP and PC3 MR cell lines once the cells underwent ∼60 HI FSS treatments over the course of ∼4–5 months of continuous culture. The sequencing was completed in collaboration with the VANTAGE core at the Vanderbilt University Medical Center (Figure 5). FASTQ sequences were aligned using Kallisto and data were processed with the Bioconductor package DESeq2 using an R kernel in Jupyter Notebook [51]. The parental, MN and MR conditions were sequenced for both the LNCaP and PC3 cell lines. We also tested a condition in which the MR cells were passaged 10 times without HI FSS exposure, abbreviated “MR*”, to examine the extent to which the cells maintain apparent shifts in their genotype. For the LNCaPs, we also sequenced “young” LNCaP MR cells (“MR‐y”), LNCaPs that have been treated with HI FSS for only ∼10 passages to examine early effects of HI FSS treatment. For the PC3 cells, we eliminated the MR‐y condition, since the PC3 cells show innate resistance to FSS, which we validated during initial studies (Figure 2), so significant genetic variations were not expected after ∼10 shear treatments. Gene Set Enrichment Analysis (GSEA) is conventionally designed to assess pairwise comparisons in data by ranking genes to a single contrast [52, 53, 54]. Since each cell type in this study consisted of three different conditions, the applicability of GSEA alone as a computational bioinformatics tool prevented definitive interpretation of the datasets from this singular platform. Thus, we further explored significantly upregulated genes that we manually filtered in our volcano plot analysis.

Euclidean distance and principal component analysis (PCA) plots confirmed that the MR cells developed distinct molecular phenotypes, in which all replicates clustered together to confirm reproducibility and accuracy (Figure S3). The Euclidean distance was calculated using the variance‐stabilizing transformed (vst) values [51]. Heatmap plots show the top 100 differentially expressed genes among the data sets for the LNCaP (Figure 5A) and PC3 (Figure 5B) cell lines. The LNCaP MN (Figure 5A) and PC3 MN (Figure 5B) conditions behaved as expected, phenotypically grouping between the parental and MR cells. The volcano plots illustrate significantly up‐ and down‐regulated genes that are evident for the MR PCa cells compared to their respective parental cells. The volcano plots in Figure 5C,D were generated using the same thresholds for significance as one another. Upregulation is defined as the log2‐fold change (L2FC) > 2, and downregulation is the L2FC < ‐2, chosen based off studies in the prior literature [52]. Significant genes were defined as those with a ‐log_10_(P_adj_) > 2 (adjusted *p*‐value (P_adj_) < 0.01). Figure S4 shows the volcano plots comparing the MN vs parental, MR vs MN, and MR* vs parental cells for the LNCaP and PC3 cell lines.

Table S1 shows the genes that are significantly expressed and up‐ or down‐regulated in the MR cells compared to both the parental and MN conditions for each cell line. The correlation of hemodynamic FSS and expression of these genes is unknown. Some of these significant genes, such as *PCDH20, EPB41L3*, and *SLC5A7* are tumor suppressors [55, 56]. We chose to further explore *CALB2*, which is significantly upregulated in the PC3 MR cells. This gene encodes for the calretinin protein and has been understudied in PCa progression. Quantitative polymerase chain reaction (qPCR) analysis confirmed significantly upregulated mRNA expression of *CALB2* in the PC3 MR cells (Figure 5E). We measured the gene expression of *CALB2* in both the LNCaP and PC3 cells to further confirm and validate the RNA sequencing results. Western blots confirmed significantly enhanced calretinin protein expression for the PC3 MR cells (Figure 5F,G). *CALB2* may also be reasonably linked to mechanotransduction‐driven cancer cell behavior, since *CALB2* buffers cytosolic Ca^2+^ levels to maintain homeostasis [57, 58]. Research suggests that increased expression of calretinin may be associated with enhancing tumor‐promoting behavior, driving increased proliferation (Figure 5H) [24, 59, 60].

The corrected total cell fluorescence (CTCF) of calretinin was measured in human tissue array samples (Figure 5I). The mean calretinin expression was highest in the adenocarcinoma and metastatic samples relative to the normal adjacent and hyperplasia/prostatitis samples. The mean expression in the adenocarcinoma samples from the prostate was 5.50 × 10^3^ ± 493, while the mean calretinin expression in the normal adjacent tissue samples was 3.25 × 10^3^ ± 2.60 × 10^3^_._ Only two metastatic samples were available, from a lymph node and rib location. The mean calretinin CTCF measured from these samples was 7.04 × 10^3^ ± 995. Figure 5J shows that across six paired patient samples calretinin expression was elevated in adenocarcinoma relative to each patient's healthy adjacent tissue, with four pairs showing an upward shift. Additionally, when grouping the mean calretinin expression by Gleason Score (Figure 5K), no statistically significant trends were observed. Representative confocal images comparing calretinin and PSMA expression in healthy adjacent tissue compared to an adenocarcinoma tissue is seen in Figure 5L.

Figure 5M shows that PCa patients with higher *CALB2* expression correlate with a lower overall survival compared to patients with lower *CALB2* expression. The survival curves show reduced probability of long‐term survival in the high‐expression group (Hazard ratio (HR) = 0.54, 95% Confidence interval (CI): 0.13–2.16, p = 0.38). In the TCGA‑PRAD cohort, mean *CALB2* mRNA expression showed no statistically significant association with Gleason score (Kruskal–Wallis, p = 0.4221; Figure 5N) [61]. *CALB2*‐high and *CALB2*‐low outliers were defined as the top and bottom 5% of *CALB2* expression, respectively, within the data set. These results show that *CALB2*‐high outliers were not restricted to the most aggressive Gleason Score. This trend is further supported by Figure S5, showing primary tumor *CALB2* expression at the patient‐level for an SUC2/PCF Dream Team Metastatic PCa cohort (Figure S5A) and the Taylor/MSKCC PCa cohort (Figure S5B) [62, 63]. Altogether, these results are consistent with the PCa microarray data in Figure 5K, where protein expression levels of *CALB2* (calretinin) showed no significant correlation with Gleason Score.

Primary tumor TCGA‐PRAD analysis was conducted, using the same threshold for *CALB2*‐high and *CALB2*‐low outliers used in Figure 5N. We examined correlation of *CALB2* expression with *DGK1* and *EPB41L3*. *DGK1* was significantly upregulated in the PC3 MR cells and *EPB41L3* was significantly downregulated in the PC3 MR cells, compared to the PC3 parental and MN conditions (Figure 5D and Table S1). *DGK1* functions to convert diacylglycerol into phosphatidic acid, to act as a molecular switch that controls lipid‐based signaling pathways and ultimately influence downstream cellular function such as cytoskeletal dynamics [64]. Primary tumor TCGA‐PRAD analysis shows a positive correlation between *CALB2* and *DGK1* expression (Figure S6A). In the primary tumor sample analysis, median *DGK1* expression was 0.8923 in *CALB2*‐high cases, compared with 0.6551 in the middle group and 0.5880 in the *CALB2*‐low cases. This association was not statistically significant (Kruskal FDR = 0.1124). However, *DGKI* showed a significant positive Spearman correlation with *CALB2* (rho = 0.1818, FDR = 0.0002) (Table S2). Co‑high enrichment was observed in 3 of 26 *CALB2*‑high tumors (odds ratio = 2.56, nominal p = 0.146) (Table S3).

We also examined the correlation between *CALB2* and *EPB41L3* expression in the TCGA‑PRAD primary tumor cohort (Figure S6B). Interestingly, *EPB41L3* primary tumor samples showed a robust and consistent association with *CALB2*‐high outliers, with a strong statistical relationship after multiple‐testing correction (Kruskal FDR = 0.0062) and a positive correlation with *CALB2* expression (Spearman rho = 0.3079, FDR = <0.0001) (Table S2). While *EPB41L3* is downregulated as a tumor suppressor in the PC3 MR cells (Table S1), it is possible that a minority of *CALB2*‑high tumors may activate compensatory or stress‑response programs that secondarily increase *EPB41L3* expression [65]. Thus, the correlated high expression pattern could represent a niche transcriptional state rather than a contradiction of the overall downregulation trend. A fraction of *CALB2*‐high outliers appears to be associated with a broader membrane/cytoskeletal expression pattern.

### 2.6 CALB2 Knockout Reduces PC3 Mechanoresistance

**FIGURE 6:**
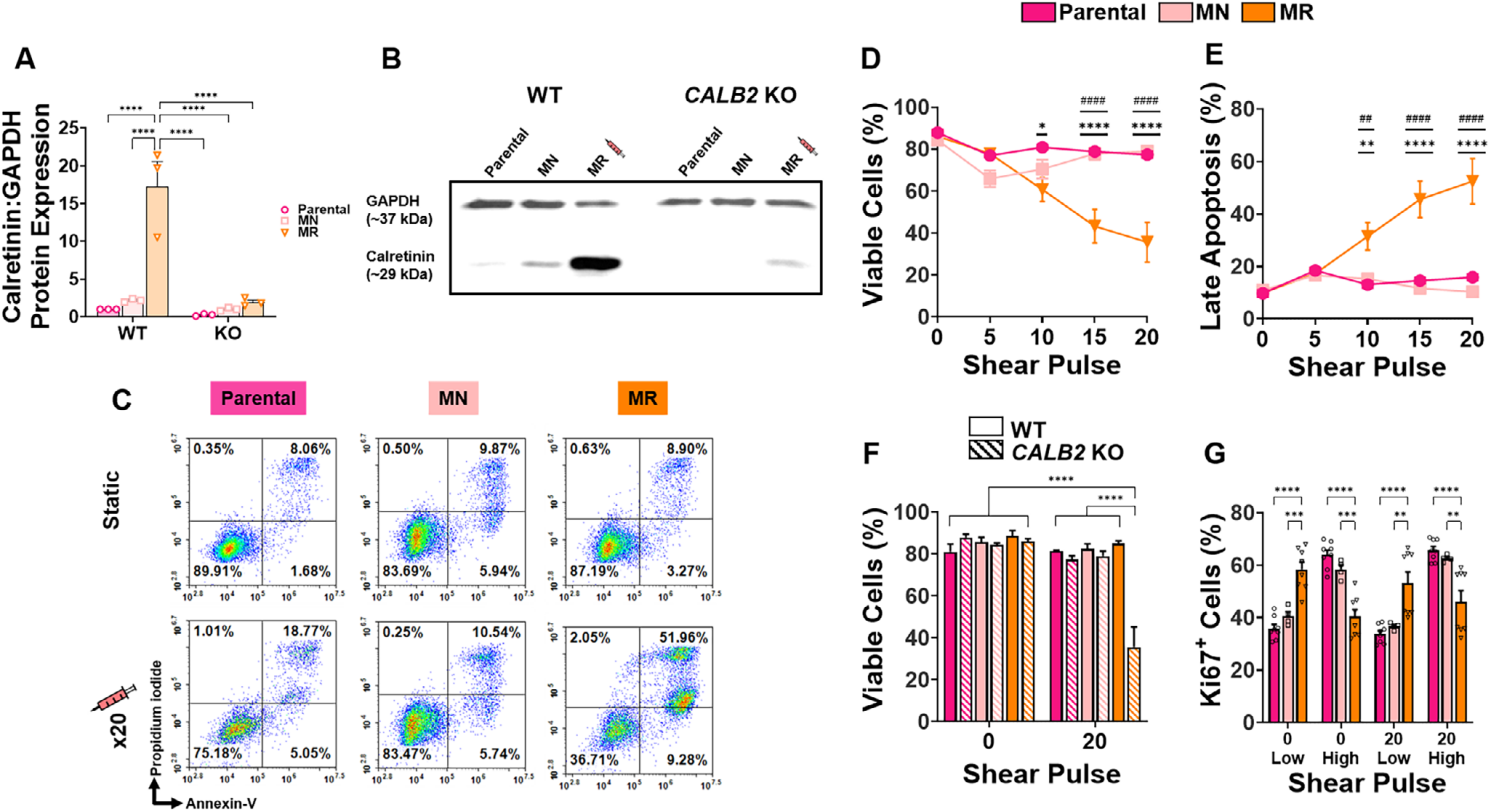
CALB2 knockout reduces fluid shear stress resistance in PC3 mechanoresistant cells. (A) Calretinin protein expression in the wild type (WT) and CALB2 knockout (KO) PC3 cells measured via Western blot, normalized to GAPDH. (B) Representative Western blot. (C) Representative flow cytometry plots for the CALB2 KO PC3 cells. Percentage of (D) viable and (E) late‐stage apoptotic PC3 cells following exposure to 20 high‐intensity fluid shear stress (HI FSS) pulses measured using the AV/PI flow cytometry assay. (F) Percentage of viable WT and KO PC3 cells under static conditions (0 pulses) and after 20 HI FSS pulses. The WT measurements are the replicates shown in Figure 2D. (G) Percentage of Ki67^+^ (low and high expression) of the PC3 CALB2 KO cells under static conditions (0 pulses) or after 20 HI FSS pulses. (A, D–F) *n* = 3 independent experiments. (G) *n* = 2 independent experiments. (A, D–G) Two‐way ANOVA. (D, E) Statistical comparison between parental and MR conditions denoted by (*) and statistical comparison between MN and MR conditions denoted by (#). */#*p* < 0.05, **/##*p* < 0.01, ***/###*p* < 0.005, ****/####*p* < 0.0001. Error bars represent mean ± SEM.

*CALB2* is significantly upregulated in the PC3 MR cells, and its expression has been used as a diagnostic marker in cancers such as mesothelioma and pancreatic cancer [59, 66, 67]. We generated *CALB2* knockout (KO) PC3 cell lines using in‐house developed lentiviral particles and confirmed that successful KO significantly decreased calretinin protein expression (Figure 6A,B). Following *CALB2* KO, we subjected the PC3 parental, MN and MR cells to 20 HI FSS pulses at the corresponding conditions associated with the data in Figure 2. The PC3 parental and MN *CALB2* KO cells showed very little changes in viability following HI FSS exposure, whereas the PC3 MR *CALB2* KO cells had significantly decreased viability following shear treatment (Figure 6C–F). After 20 shear pulses, the viability of the PC3 MR *CALB2* KO cells decreased to 35.63 ± 9.57%, and was 77.43 ± 1.71% and 79.03 ± 2.39% for the parental and MN *CALB2* KO conditions, respectively. Additionally, significant differences were observed in the viability of the PC3 MR cells between the wild type (WT) and KO conditions in Figure 6F at 20 shear pulses, while the PC3 parental and MN conditions show insignificant differences between their respective WT and KO conditions. This indicates that *CALB2* contributes significantly to the acquired molecular phenotypic changes observed in the MR PC3 cells. This study represents the first of its kind to explore potential links between *CALB2* (calretinin) and FSS in cancer.

Proliferation in the PC3 *CALB2* KO cells was measured following 20 HI FSS treatments. Similar trends were observed following 0 (static) and 20 shear pulses for the parental and MN *CALB2* KO conditions, in which the fraction of cells in the low and high Ki67^+^ readout were largely unchanged. Interestingly, the MR *CALB2* KO condition overall showed the opposite trend, with a larger Ki67^+^ fraction observed to have low Ki67^+^ expression relative to high expression. Lower Ki67 expression may indicate decreased cell cycle activity or lower transcriptional activity. 24 h following exposure to 20 HI FSS pulses, the Ki67^+^ fraction of MR *CALB2* KO cells with low Ki67^+^ expression was 53.1 ± 4.35%, while the fraction of cells with high Ki67^+^ expression was 46.1 ± 4.24%. These results align with the reduced viability of MR *CALB2* KO cells after HI FSS exposure, as the increased proportion of cells in the low Ki67^+^ category, even at the static conditions, indicates a shift toward diminished cell‑cycle or transcriptional activity relative to the parental and MN *CALB2* KO conditions.

### 2.7 Orthotopic Prostate Cancer Study

We first conducted an in vivo pilot study in which subcutaneous tumors were inoculated on the flanks of six to eight‐week‐old male NU/NU mice. These results showed that the LNCaP and PC3 MR cells developed into higher tumor burden compared to the parental cells (Figure S7). As a follow‐up study to examine detectable changes in metastatic progression using a more clinically relevant model, we performed an orthotopic PCa study. We completed this study using the PC3 cells since these cells are androgen‐independent and represent advanced disease [68].

**FIGURE 7:**
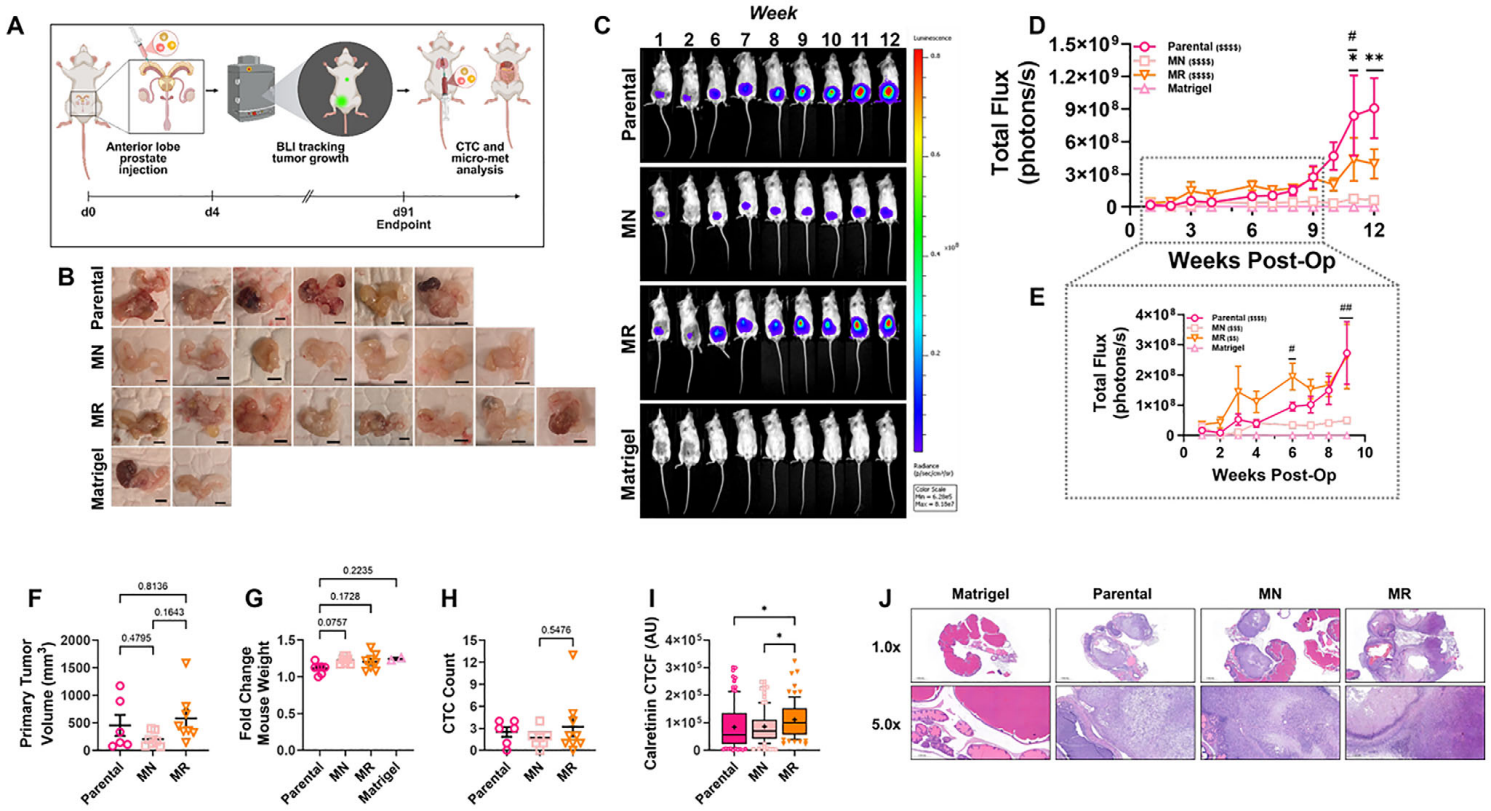
Orthotopic prostate cancer model reveals similar growth between parental and mechanoresistant cells. (A) Timeline for in vivo orthotopic study. (B) Photographs of primary tumors following resection at d91, scale bar = 5 mm. (C) Representative bioluminescence (BLI) images of the parental, MN, MR, and Matrigel conditions inoculated in the anterior lobe of the mouse prostate. (D) Average total flux of the primary tumor region measured from the BLI images of the mice. (E) Same average total flux as shown in (D), zoomed in to examine 1–9 weeks post‐op. (F) Volume of the primary tumor measured at endpoint using calipers (Equation 5). (G) Fold change in the mouse weight. (H) Circulating tumor cell count identified as mCherry^+^ cells using flow cytometry. (I) Calretinin protein expression measured via immunofluorescence in the primary tumor tissue samples. *n* = 7–8 mice per condition with *n* = 2 mice for the Matrigel control. (J) Representative H&E images of the primary tumor for each condition, 1.0X scale bar = 2 mm and 5.0X scale bar = 0.50 mm. (D,E) Two‐way ANOVA, (#) represents the comparison between the MR and MN condition and (*) represents the comparison between the MR and parental condition. (F‐I) One‐way ANOVA. (I) Box plots showing the 10–90th percentile and (+) indicates the mean. */#*p* < 0.05, **/##*p* < 0.01, ***/###*p* < 0.005, ****/####*p* < 0.0001. (D,E) Simple linear regression to confirm significant deviation from zero, $*p* < 0.05, $$*p* < 0.01, $$$*p* < 0.005, $$$$*p* < 0.0001. Error bars represent mean ± SEM.

1 × 10^6^ PC3 cells were surgically inoculated into one anterior lobe in six to eight‐week‐old male, NOD/SCID mice (Figure 7). We chose the anterior lobe since it allows for a larger injection volume and is one of the most common injection sites for developing orthotopic PCa models due to its proximity to the seminal vesicles and thus represents greater physiological relevance [69, 70]. We monitored tumor progression over time via bioluminescence imaging (BLI) until a humane endpoint was reached at 12 weeks (Figure 7A–E). The total flux remained lowest for the PC3 MN condition, confirmed by visually lower BLI signals of the primary tumor and a smaller primary tumor volume measured at endpoint (204.0 ± 50.4 mm^3^) (Figure 7F). The PC3 parental and MR conditions showed comparable BLI readouts for the first nine weeks post‐op, where the average BLI was higher for the PC3 MR mice up to this point (Figure 7E). After nine weeks, the PC3 parental mice showed a more rapid increase in BLI (Figure 7D). The average primary tumor volume of PC3 parental and MR samples was 454.7 ± 190.0 and 581.6 ± 160.1 mm^3^ at endpoint, respectively.

At endpoint, we measured the volume of the primary tumors using calipers following resection (Figure 7F) and recorded the fold change in mouse weight (Figure 7G). Non‐significant changes in mouse weight were observed among all four conditions, showing that all of the mice gained weight due to increased tumor burden and healthy diets with age (Figure 7G). Overall, little metastasis was observed, likely because we used Matrigel to help the primary tumors engraft, which may provide extra resistance for tumor cells to navigate out of the primary tumor region. However, few CTCs were detected (Figure 7H). The average CTC count was 2.50 ± 0.67 CTCs for the parental condition, 1.71 ± 0.47 CTCs for the MN condition and 3.22 ± 1.33 CTCs for the MR condition. It is also likely that some micrometastases may have avoided detection in BLI due to the strong signals emitted by the primary tumors. However, upon dissection of the kidney, lungs, and liver at endpoint, very few macrometastases were observed. These results are consistent with a prior study by Kim et al., who inoculated PC3 tumors on the dorsal prostate of BALB/c mice and observed only primary tumor BLI signal [50].

Calretinin protein expression in the primary tumor samples was measured using immunofluorescence (Figure 7I). The expression was highest in the MR condition (1.11 × 10^5^ ± 7.56 × 10^3^) compared to the parental (8.41 × 10^4^ ± 7.67 × 10^3^) and MN (8.64 × 10^4^ ± 5.72 × 10^3^) conditions, consistent with increased calretinin expression observed in the PC3 MR cell in vitro.

Representative hematoxylin and eosin (H&E) images show the distinct morphological differences observed between the Matrigel control conditions and the primary tumor regions in the parental, MN, and MR conditions (Figure 7J). The parental conditions show a relatively compact tumor environment while the MR tumor shows more heterogenous tissue density.

## 3 Discussion

Mechanoadaptation in cancer is an emerging yet complex field of study. It encompasses a sophisticated set of processes that varies between cell type and cancer type. Even within a single patient, each CTC has its own phenotypic and molecular signatures that support its survival under dynamic physiological conditions. In our study, the impact of HI FSS on tumor cell viability revealed cell‐specific responses (Figure 2). The LNCaP cells showed more necrosis than apoptosis in response to HI FSS exposure at 4 (Figure 2B,C) and 24 h (Figure S1A,B) timepoints. This is likely because the LNCaP cells are innately more vulnerable to harsh or extreme conditions due to their lymph node origin and epithelial‐like phenotype [27, 28, 29]. Interestingly, a study by Fan et al. showed that HCT116 colorectal cancer cells counterintuitively maintained higher cell viability with increased fluid deformation, undergoing 20 h of FSS exposure within a microfluidic circulatory pump [18]. Increased survival was correlated to upregulated expression levels of oncoproteins β‐catenin and c‐Myc, so it is possible that the LNCaP cells are undergoing a similar response. The LNCaP cells are AR‐dependent, and overexpression of the AR has been linked to Wnt/β‐catenin transcriptional activity [18, 71]. We observed no changes in PC3 viability following HI FSS exposure for any of the conditions tested (Figure 2D,E, Figure S1C,D). This indicates that the immortalized PC3 cells are innately resistant to 3950 dyn/cm^2^ of HI FSS, consistent with previous reports [9, 10, 11]. Our HI FSS system uses 30 G needles, and the needle diameters are still much larger than the size of an artery or a vessel, therefore, the cells are not experiencing capillary‐like squeezing deformation within the needle during each shear pulse.

Throughout these studies we observed more significant changes in the phenotype and genotype of the PC3 MR cells compared to the parental and MN conditions, whereas the LNCaP MR cells maintained similar phenotypic characteristics to the parental cells, despite the vast differences observed in viability following HI FSS exposure. There are very few studies that have measured changes in cancer cell size following LI or HI FSS exposure. One study by Barnes et al. observed that HI FSS exposure had no significant effect on tumor cell size after a single exposure to 10 HI shear pulses [9]. The LNCaP and PC3 MR cells showed a decrease in cell size in suspension under extremely LI FSS (66 µL/min) when passing through the flow cell (Figure 3B). This could suggest that changes in cell size in fluid flow could be correlated with cytoskeletal rearrangement to mediate cell stiffness and deformation in response to fluid flow, which is beyond the scope of the current study [9, 72, 73]. Considering that the LNCaP parental and MR cells showed non‐significant morphology variations when comparing the two (Figure 3B,D), this suggests that the LNCaP MR cells can possibly reset their phenotype once recovered from HI FSS treatment. In the LNCaP MR cells, cellular morphology and EMT characteristics were not directly related since the LNCaP MR and parental cells showed similar morphologies, yet the LNCaP MR cells had a significantly reduced E‐cadherin expression relative to the parental cells (Figure S2A). Since vimentin expression was undetected in the LNCaP parental, MN and MR cells, we are unable to conclude that the MR condition is shifting toward an EMT‐like phenotype but this could still suggest a hybrid EMT phenotype. Future studies could examine the expression of additional mesenchymal markers, such as N‐cadherin, to better understand this adaptation [41, 74].

The PC3 MR cells displayed a significantly reduced cell area and decreased circularity when adhered (Figure 3E), compared to the PC3 parental and MN conditions, consistent with a decreased cell size in suspension (Figure 3C). This could potentially indicate that the PC3 MR cells undergo mesenchymal‐to‐amoeboid transitions (MAT) to adapt into higher plasticity, as this could explain the smaller cell sizes observed [74]. Amoeboid cells have shown increased metastatic capacity in various cancer types, including PCa [40, 75]. This is consistent with our EMT analysis, which revealed that the PC3 MR cells show a hybrid EMT phenotype compared to the parental cells, with an EMT ratio between the PC3 parental and MN conditions (Figure S2B,C). These results align with previous studies that exposed tumor cells to LI FSS [43]. This is not surprising given that clinically, the histology of most metastatic tumors show an epithelial phenotype, suggesting EMT reversion occurs during metastasis, and many tumor cells metastasize in a hybrid or partial EMT state, since both states confer advantages toward cancer cell survival at different stages in the metastatic cascade [39, 41, 76, 77].

Increasing efforts have worked to understand how FSS impacts cancer cell proliferation in cancer‐promoting and cancer‐inhibiting ways [21]. Our previous research has shown that exposure of colorectal cancer cells to 10 pulses of HI FSS induced no significant changes in the percentage of proliferating cells [21]. Additionally, most research has shown that LI and HI FSS cause cell cycle arrest and promote cell death through stress‐induced mechanisms such as decreased expression of cyclins (i.e. cyclins D and E) or smad5 activation [48, 78, 79]. The LNCaP cells showed lower proliferation levels than PC3 cells, which is expected based on the nature of the cell lines (Figure 4A,C) [27, 28, 29, 80]. The increased Ki67 MFI observed in the LNCaP and PC3 MR conditions (Figure 4A,C) did not show direct correlation with stages of the cell cycle (Figure 4B,D), except for the PC3 MR cells 24 h post‐HI FSS exposure. The PC3 MR 24 h condition showed a significantly higher S phase fraction and significantly lower G_1_ fraction, consistent with increased Ki67 expression, suggesting increased proliferative activity with accelerated G_1_‐S phase transition. We chose to measure the cell cycle phases for the LNCaP MR cells 24 h post‐shear (Figure 4B) to compare to the PC3 results. Interestingly, a similar yet more subtle trend was observed, where the LNCaP MR cells 24 h post‐HI FSS shifted toward an accelerated G_1_‐S phase transition. Altogether, neither the LNCaP nor PC3 cells reached cell cycle arrest, as no stages in the cell cycle readout trended to show a notable population shift in a single phase (Figure 4B,C). This is significant because in previous studies, FSS treatment resulted in the opposite effect and tumor cells underwent arrest [48, 78, 79]. Therapeutically, this underscores the value of future interventions aimed at re‐establishing checkpoint regulation when targeting tumor cells in the circulatory system.

The LNCaP and PC3 MR cells showed significantly increased Piezo1 expression relative to the parental and MN cells (Figure 4F–H). Increased Piezo1 expression levels in cancer cells has been correlated with many cancer‐promoting responses, such as tumor cell motility, migration, and proliferation [49, 50, 81, 82]. Additionally, FSS has been shown to increase Piezo1 expression levels in cancer cells [50]. Previous research has shown that enhanced cell cycle activity (i.e., increased S phase) was consistent with significantly increased Piezo1 expression, while also increasing YAP/TAZ nuclear localization [83]. We observed positive correlation between the percentage of LNCaP cells in S phase and Piezo1 expression (Figure 4I), as well as among the Ki67 MFI and Piezo1 expression in the LNCaP and PC3 cells (Figure 4I,J). Future studies could introduce GsMTx‐4 as a Piezo1 inhibitor to gain additional mechanistic insight and further understand factors that drive increased Piezo1 expression, or explore additional MSCs such as transient receptor potential (TRP) channels [8, 29, 84, 85, 86]. The use of GsMTx‐4 was prohibitively expensive for in vivo use. Our lab has also shown that FSS activates Piezo1 in cancer cells, and that we can take advantage of this to induce cancer cell apoptosis by combining mechanical stimuli with anti‐cancer drugs such as TRAIL [8, 21, 29, 84, 85]. So, elevated Piezo1 expression could indicate that the MR cells are more TRAIL‐sensitive following FSS exposure.

Bulk RNA‐sequencing revealed that the MR cells demonstrate unique molecular phenotypes, distinct from the parental and MN cells (Figure 5). Filtering the significantly up‐ and down‐regulated genes within the MR cells compared to the parental and MN conditions showed that the LNCaP and PC3 MR cells adopted distinct genetic signatures. Since GSEA was developed to process pairwise data comparisons, using this tool was not appropriate to definitively delineate biological processes associated with the MR cells, and so we chose to focus on the significant genes we filtered (Figure 5C,D). For instance, *RND3* is significantly upregulated in LNCaP MR cells, and studies have indicated that *RND3^−^* cancer patients show greater survival than *RND3^+^* patients, which could indicate that the MR LNCaP cells have developed a more aggressive phenotype [87, 88]. We further explored the therapeutic implication of *CALB2* in the PC3 MR cells based on its biological functions and potential roles in mechanotransduction, which remains largely unexplored. Prior work has not examined *CALB2* or calretinin in metastatic PCa, and *CALB2*‐high expression outliers have not been thoroughly investigated or characterized in this context, to delineate its relationship with other mechanoadaptive genes or lineage markers. Additionally, the relationship of *CALB2* to FSS exposure in cancer metastasis remains unaddressed.

Increased calretinin expression was observed in human adenocarcinoma and metastatic tissue samples compared to normal adjacent samples, as well as patients who presented symptoms such as hyperplasia and prostatitis but did not present PCa (Figure 5I). These observations suggest that calretinin levels may vary with disease state in some patients, but the biological significance of this variation remains unclear. This trend is further supported by Figure 5J, in which four out of six patients showed increased calretinin levels in adenocarcinoma samples next to normal adjacent tissue.

Multiple clinical PCa datasets show that *CALB2* expression does not correlate with Gleason Score in patients (Figure 5N and Figure S5). However, these data showed that *CALB2*‐high outliers were repeatedly observed across datasets and across Gleason Scores, indicating that expression might be patient‐specific rather than directly reflecting cancer aggressiveness or lineage. Additionally, elevated *CALB2* expression correlated with increased *DGKI* and *EPB41L3* levels, suggesting a reproducible co‑expression pattern that reflects underlying molecular heterogeneity within PCa. The downregulation of *EPB41L3* in PC3 MR cells (Table S1) contrasts with its higher expression in *CALB2*‑elevated tumors, suggesting that *EPB41L3* participates in a heterogeneity‑associated transcriptional program in patient samples that is distinct from the mechanoresistant state modeled in PC3 MR cells. Our results showed that *CALB2* KO significantly reduced the viability of the PC3 MR cells when re‐exposed to HI FSS up to 20 shear pulses (Figure 6). Since the PC3 parental and MN *CALB2* KO cells showed no significant shifts in viability following HI FSS treatment, we may conclude that *CALB2* plays a major role in the survival of PC3 MR cells subjected to harsh biophysical forces. Additionally, the MR *CALB2* KO cells show less proliferative activity compared to the parental and MN *CALB2* KO conditions (Figure 6G), consistent with the reduced viability observed in the MR *CALB2* KO cells. Since *CALB2* (calretinin) has been rarely studied in cancer, little is known about its interactions with downstream signaling pathways, such as those that interact with *DGK1*. To our knowledge, there are no studies that have examined the role of calretinin in mechanotransduction responses to mechanical stimuli. Overexpression of calretinin in malignant mesothelioma led to increased activation of the FAK signaling pathway, due to calretinin‐induced upregulation of FAK levels [24]. Additionally, previous knockdown of *CALB2* in pancreatic ductal adenocarcinoma (PANC‐1 cells) led to a significant reduction in cell viability, while diminishing the invasion and migration potential [59].

Calretinin buffers intracellular Ca^2+^ levels, so future studies could treat PC3 MR *CALB2* KO cells with MSC inhibitors to inhibit Ca^2+^‐gated channels such as Piezo1. Previous studies in our lab have shown that Piezo1 is activated in cancer cells via mechanical stimuli, including FSS [8, 10, 21]. Since calretinin exists in the cytosol, this would delineate if calretinin is the primary intracellular Ca^2+^ chelator following intracellular influx. It is also possible that the higher Piezo1 expression observed in the PC3 MR cells could promote this response, in which elevated calretinin expression could help maintain cellular homeostasis with more Piezo1 channels available for activation [85, 89]. Mechanistic investigations of this nature would also provide insight regarding synergistic apoptotic mechanisms, such as combination treatments with TRAIL [8, 10, 21, 84]. Given the lack of documented small‐molecule inhibitors that directly target calretinin due to the lack of well‐defined ligand‐binding pockets, understanding its downstream signaling mechanisms in metastatic PCa may help identify more tractable therapeutic targets or approaches. Since calretinin is part of a broader network of Ca^2+^‐binding proteins, targeting it alone may be insufficient due to potential compensatory activity from proteins such as calmodulin. Thus, future studies would need to further explore this phenomenon prior to clinical translation.

We first conducted a pilot, subcutaneous in vivo study, where we inoculated tumors on the flanks of immunodeficient, male NU/NU mice (Figure S7). We observed that the MR cells produced a higher tumor burden than the parental cells. This seemingly contradicts a previous study in our lab, which showed that DU145 and LNCaP PCa flank tumors inoculated on NU/NU mice immediately following a one‐time exposure to 10 HI FSS pulses, and observed that the static conditions grew larger tumors than the shear conditions [11]. However, our previous study did not explore the effects of repeated shear exposure on the inoculated cells, but instead examined the effects of one HI FSS treatment on the parental cells that have not previously undergone sustained shear treatment. Therefore, these differences affirm that the observed phenotypic shifts that the MR cells acquired were maintained in vivo.

As a follow‐up, we completed an orthotopic PCa study that more accurately recreates the local environment and allows for the possibility of bloodborne metastasis [68]. After inoculating tumors into the anterior lobe of the prostate, we observed the PC3 parental and MR conditions demonstrated the highest tumor burden as evident by the BLI and primary tumor volume at endpoint (Figure 7C–F). The PC3 MN tumor burden remained lowest overall, with the smallest primary tumors at endpoint. The lower tumor burden evident for the PC3 MN condition is attributed to higher passage number (longer culture time) since these cells were not modified in any other way from the PC3 parental condition. The PC3 MR tumors appeared to grow slightly faster than the PC3 parental tumors for the first nine weeks of the study. This could be attributed to the enhanced proliferation observed in Figure 4C. It also appears that the PC3 parental tumors reached their exponential growth phase right at nine weeks (Figure 7D,E), whereas the PC3 MR tumors did not reach this point during the study. Higher levels of calretinin were measured in the PC3 MR tumor samples (Figure 7I). However, the magnitude of this difference was more subtle than in vitro, possibly reflecting context‑dependent variability within the in vivo tumor microenvironment rather than a uniform mechanoadaptive response.

Little metastasis was observed in all conditions, which is consistent with previous studies that developed PCa models using the anterior and dorsal prostate lobe [50, 90]. The lung is a common organ for metastases to occur in mouse studies, so it is possible we did not observe any due to the severe immunodeficiency presented by the NOD/SCID mice and lack of a welcoming environment to promote tumor cell arrest and growth [90]. To fully understand and study mechanoresistance in advanced PCa in vitro, the FSS parameters extended beyond the physiological range so that significant differences in PC3 response can be accessed.

## 4 Conclusions

This work represents the first attempt to develop a stable MR cancer cell line. Herein, we have begun to unravel the survival strategies developed by CTCs in the bloodstream while under physiological FSS during metastasis. Metastasis is a complex and well‐studied process, but many gaps in knowledge persist, particularly in the circulatory stage of this process. These findings are significant, as we are the first to develop MR cells through repeated HI FSS treatment over the course of 4∼5 months. We performed a thorough characterization of the phenotypes adapted by the MR cells compared to their respective parental and MN counterparts. Elucidating these characteristics provides insight into how mechanically adapted tumor cells survive metastatic stress, offering a deeper understanding of this aggressive cell population. RNA‑sequencing identified several candidate genes, including *CALB2*, that may contribute to mechanoadaptive or stress‑responsive programs and warrant further investigation, consistent with the distinct transcriptional patterns observed in *CALB2*‑elevated tumors. Additionally, the orthotopic model demonstrated that MR cells remain tumorigenic in vivo, with calretinin levels showing similar patterns to those seen in vitro. This model thus offers a foundation for future work dissecting mechanoadaptive programs in vivo. Altogether, these findings have implicated the mechanoadaptive mechanisms of CTCs in PCa that will lead to more effective therapies to disrupt the metastatic spread of cancer.

## 5 Experimental Section

### 5.1 Cell Culture

Human prostate adenocarcinoma cell lines PC3 (ATCC #CRL‐1435) and LNCaP (ATCC #CRL‐1740) were purchased from American Type Culture Collection (Manassas, VA, USA). LNCaP and PC3 cells were cultured in RPMI 1640 cell culture medium, supplemented with 10% (v/v) fetal bovine serum (Gibco), 1% (v/v) penicillin‐streptomycin (PenStrep) (Fisher Scientific, Carlsbad, CA, USA), and 10 mM HEPES (v/v) (Fisher Scientific). LNCaP media was also supplemented with 1 mM sodium pyruvate (Fisher Scientific). Cells were incubated under humidified conditions at 37°C and 5% CO_2_ and did not exceed 90% confluence. Cells were regularly mycoplasma tested every 2–3 months.

LNCaP and PC3 cells were washed in Hank's Balanced Salt Solution (HBSS) free of Ca^2+^ and Mg^2+^ (HBSS‐/‐) (Corning, Manassas, VA, USA). LNCaP cells were then treated with Accutase (Sigma‐Aldrich, St. Louis, USA) and PC3 cells were treated with 0.25% trypsin‐EDTA (Gibco) for 5–6 min at 37°C. Cells were centrifuged at 300 x *g* to remove dissociation agent and resuspended in complete media as needed for experimentation.

### 5.2 Fluid Shear Stress Treatment of Mechanoresistant Cells

Cells were lifted as described in the “Cell Culture” section and then resuspended in complete media at a concentration of 3 × 10^5^ cells/mL prior to exposure to fluid shear stress.

The fluid shear stress (FSS) that a cell experiences in the 30 G needle was estimated using Poiseuille's equation:

(1) $$\tau_{\max}=\frac{4Q\mu }{\pi R^{3}}$$

where τ is the wall shear stress (dyn/cm^2^), *Q* is the flow rate (cm^3^/s), i.e., 18 mL/min, μ is the viscosity of the RPMI medium, which is assumed to be water at room temperature (RT) and standard pressure (0.01 dyn*s/cm^2^), and *R* is the inner radius of the 30 G needles (7.94 × 10^−3^ cm). The maximum FSS (5920 dyn/cm^2^) is located at the wall of the conduit since the local FSS varies linearly with radial position. The area‐averaged FSS is equal to two‐thirds of the maximum (3950 dyn/cm^2^). A single pulse through the needle has a transit time of 1.08 ms and each shear pulse was administered every two min. The LNCaP cells were exposed to 0–10 shear pulses and the PC3 cells were exposed to 0–20 shear pulses per treatment. Following FSS exposure, the MR cells were seeded and placed back into the incubator at 37°C and 5% CO_2_. Media was changed 24 h following FSS treatment.

The minimum FSS can be estimated using the following equation:

(2) $$\tau_{\min}=\tau_{\max}\;\left(\frac{r}{R}\right)$$

where *r* is the radius of the cell, estimated in this case to be 9.31 µm [9, 32]. The Reynolds number for this flow was calculated to confirm laminar flow and justify the use of Poiseuille's equation, and is defined as follows:

(3) Re=ρvDμ

where ρ is the buffer density, assumed to be water at standard RT and pressure (0.998 g/cm^3^), *v* is the flow velocity, *D* is the inner needle diameter and μ is the buffer viscosity. The Reynolds number at a flow rate of 14 mL/min is 1850. This value is below 2200, the threshold for laminar flow, suggesting that Poiseuille's equation is appropriate to predict the average FSS.

### 5.3 Cell Morphology and Phase Contrast Imaging

When cultured cells were at 50%~60% confluency in a T182 flask so that individual cells can be outlined, 20X phase contrast images were captured using an Olympus IX81 microscope equipped with a 20X objective. Cell morphology was analyzed using a macro in FIJI [36].

### 5.4 Western Blotting

#### 5.4.1 Calretinin Lysate Preparation

PC3 cells were lifted as described in the “Cell Culture” section. Samples were prepared containing 5 × 10^5^ cells each and were lysed in 4X Laemmli sample buffer (1610747, BIO‐RAD, Hercules, CA, USA), prepared following the manufacturer's directions. The lysate samples were then briefly sonicated.

#### 5.4.2 Vimentin and E‐Cadherin Lysate Preparation

5 × 10^5^ LNCaP and PC3 cells were plated in wells of a 6‐well dish (CellTreat) and maintained at 37°C and 5% CO_2_ overnight. The next day, samples were lysed in freshly prepared lysis solution using a cell scraper. The Blue Loading Buffer Pack (7722, Cell Signaling Technology) was used to prepare the lysis buffer according to the manufacturer's instructions. The samples were transferred to microcentrifuge tubes and briefly sonicated.

#### 5.4.3 SDS Gel Electrophoresis

The materials used to prepare the gels were: sodium dodecyl sulfate (SDS) (L4509, Sigma‐Aldrich), ammonium persulfate (1610700, BIO‐RAD), TRIS base (17926, Thermo Scientific), TEMED (17919, Thermo Scienific), 30% acrylamide/bis solution 37.5:1 with 2.6% crosslinker (1610158, BIO‐RAD, Hercules, CA, USA), and HCl (60‐007‐48, Fisher Scientific). The materials used to prepare the 10X running buffer included: glycine (G8898, Sigma‐Aldrich, St.Louis, MO, USA) and the same TRIS base used for the gels. The 1X TBS‐t solution was prepared by diluting the 10X TBS (46‐012‐CM, Corning) in Milli‐Q (MQ) water and adding 0.1% Tween‐20.

Samples were warmed at 90°C for 10 min, followed by brief centrifugation. Next, 20 µL of sample and 5 µL of Precision Plus Protein All Blue Standards (1610373, BIO‐RAD) were loaded per lane into a 10% sodium dodecyl sulfate (SDS)‐polyacrylamide gel. The gel was run at 100 V in a 1X running buffer, and protein was transferred using the Trans‐Blot Turbo Transfer System (1704157, BIO‐RAD) with the Trans‐Blot Turbo Midi 0.2 µm PVDF Transfer Packs, following the manufacturer's instructions (1704157, BIO‐RAD).

Membranes were activated in 100% methanol (K977, VWR) for 30 s while rocking, and washed 3x in 1X TBS‐t for 5 min each, and the membranes remained on the rocker for all subsequent steps. Following washes, membranes were blocked for 1 h at RT. Primary antibodies were incubated in blocking solution overnight at 4°C. For the blocking steps, the calretinin membranes were blocked in LI COR BIOTECH LLC Intercept (PBS) Blocking Buffer (92770001, LI COR BIOTECH), and the vimentin and E‐cadherin membranes were blocked using 5% (w/v) nonfat dry milk/0.1% TWEEN 20 (P6586, Millipore Sigma) in 1X TBS. The dried milk was purchased from Cell Signaling Technology (9999).

#### 5.4.4 Antibody Staining

**Calretinin**: PC3 membranes were stained with calretinin (E7R60) XP Rabbit mAB primary antibody, diluted at 1:1000 (92635, Cell Signaling Technology). **Vimentin and E‐cadherin**: Membranes were stained with anti‐vimentin (1:1000) (V6630, Sigma‐Aldrich) and E‐cadherin (1:500) (3195, Cell Signaling Technology) diluted in blocking solution.

The next day, membranes were washed 3x in 1X TBS‐t for 5 min each. **Calretinin**: hFaB Rhodamine Anti‐GAPDH Primary Antibody (1:1000) (12004167, BIO‐RAD) and StarBright Blue 700 Goat Anti‐Rabbit IgG secondary (1:2000) (12004161, BIO‐RAD) were diluted in blocking buffer for 1 h at RT. **Vimentin and E‐cadherin**: Secondary antibodies were diluted in blocking buffer; anti‐GAPDH‐rhodamine (1:1000), StarBright Blue 700 Goat Anti‐Rabbit IgG secondary (1:2000) and StarBright Blue 520 Goat Anti‐Mouse IgG secondary (1:2000) (12005866, BIO‐RAD). Membranes were imaged using a ChemiDoc MP Imaging System (BIO‐RAD) and protein bands were quantified using an in‐house macro developed in FIJI [36].

### 5.5 Annexin‐V/propidium Iodide Flow Cytometry Assay

Cellular viability, apoptosis and necrosis was measured using FITC‐conjugated annexin‐V (AV) (BD Pharmingen, San Diego, CA, USA) and propidium iodide (PI) (BD Pharmingen). The manufacturer's instructions were followed to prepare samples for flow cytometry analysis.

For the initial viability studies in Figure 2, the LNCaP and PC3 cells were processed 4 and 24 h post‐HI FSS exposure following incubation at 37°C and 5% CO_2_. For the *CALB2* KO experiments in Figure 6, PC3 cells were assessed 24 h following FSS exposure. Media was transferred from 12‐well plates (CellTreat) to microcentrifuge tubes. Cells were detached from the well plate by incubating with 0.25% trypsin‐EDTA for the PC3 cells and Accutase for the LNCaP cells at 37°C for 6 min. Complete media was added to neutralize the solution and collect the samples from each well to transfer to the respective microcentrifuge tubes. Cell samples were centrifuged at 300 x *g* for 5 min and washed once in HBSS‐/‐. Next, cells were incubated in the AV (2:100 dilution) and PI (3:100 dilution) dyes in HBSS with Ca^2+^ and Mg^2+^ (HBSS++) in the absence of light for 15 min. Cells were analyzed using the NovoCyte Quanteon (Agilent) flow cytometer. Viable cells are AV‐/PI‐, early apoptotic cells are AV+/PI‐, necrotic cells are AV‐/PI+ and late apoptotic cells are AV+/PI+. Single‐stained samples were used for each experiment to calibrate the instrument.

### 5.6 Ki67 Flow Cytometry Assay

LNCaP MR and PC3 MR cells were exposed to HI FSS and incubated at 37°C and 5% CO_2_ in a 24‐well plate (CellTreat) for the 4 and 24 h timepoints. To measure cell proliferation, samples of 5 × 10^5^ cells were washed once in HBSS++ at 300 x *g* for 5 min. Next, samples were incubated for 30 min at RT in the dark in the LIVE/DEAD Fixable Violet Dead Cell Stain (L34955, Invitrogen) diluted at 1:1000 in HBSS++. Following incubation, cells were washed once in HBSS++ at 300 x *g* for 5 min. Samples were resuspended in 500 µL of 4% paraformaldehyde (PFA) (v/v) (15714S, Electron Microscopy Sciences) for 15 min at RT to allow cells to become fixed. 1 mL of HBSS++ was added and cells were washed twice in 1% (w/v) bovine serum albumin (BSA) (A1470, Millipore Sigma), centrifuged at 800 x *g* for 5 min. Cells were permeabilized in 90% ice‐cold methanol by adding 900 µL of methanol dropwise to 100 µL of HBSS++ for 10 min on ice. After incubation, samples were washed twice in 1% BSA at 800 x *g* for 5 min. Samples were incubated in 100 µL of staining solution of either PE anti‐human Ki‐67 antibody (350504, BioLegend) diluted to 3:100 or the PE Mouse IgG1, κ Isotype Ctrl antibody (MOPC‐21) (400112, BioLegend) diluted to 1.5:100 in 1% BSA for 45 min in the dark at RT. After incubation, 1 mL of 1% BSA was added to the samples, and the samples were washed twice in 1% BSA at 800 x *g* for 5 min. Samples were analyzed using a NovoCyte Quanteon Flow Cytometer using the V445 and Y586 parameters.

For the proliferation studies in the PC3 *CALB2* KO cells, the experiments followed the same protocol as above with a few minor changes. Ki67 in static (0 pulses) or 24 h following 20 shear pulse treatments were measured. Cells were stained with the LIVE/DEAD Fixable Violet Dead Cell Stain, then fixed and permeabilized. Next, samples were incubated for 30 min with Ki‐67 Monoclonal Antibody (SolA15), eFluor 660, eBioscience (50‐5698‐82, BioLegend) at a 0.8:100 dilution in 1% BSA. After incubation, samples were washed and analyzed using either a FACS SONY MA900 sorter managed by the Shared Equipment Authority at Rice University, or a Guava easyCyte 12HT benchtop flow cytometer (MilliporeSigma, Burlington, MA, USA).

### 5.7 Cell Cycle Flow Cytometry Assay

LNCaP MR and PC3 MR cells were exposed to HI FSS and incubated at 37°C and 5% CO_2_ in 24‐well plates for the 4 and 24 h timepoints. The parental, MN and MR samples were lifted from culture as described in the “Cell Culture” section. 3 × 10^5^ cells per sample were collected in a 1.5 mL microcentrifuge tube. The samples were washed once in HBSS++ 300 x *g* for 5 min. Then, the samples were stained with the LIVE/DEAD Violet Viability/Vitality Kit, for 405 nm excitation (L34958, Invitrogen) diluted 1:1000 in HBSS++. Samples were incubated at RT in the dark for 30 min and then were washed once in HBSS++ at 300 x *g* for 5 min. samples were fixed in 70% ice‐cold methanol, added dropwise while vortexing, and were stored at −20°C for at least 2 h before further processing.

Prior to PI staining, samples were allowed to equilibrate to RT. Samples were centrifuged at 500 x *g* for 5 min. Next, the cells were washed one time in 1X phosphate buffered saline (PBS) without Mg^2+^ and Ca^2+^ (PBS‐/‐) (Corning), 500 x *g* for 5 min. The cell pellets were gently resuspended in 200 µL of 1X PI+RNase staining solution using the Propidium Iodide Flow Cytometry kit (ab139418, Abcam), and were incubated at 37°C for 30 min. Samples were then transferred to a 96‐well plate (Greiner Bio‐One) and analyzed using a NovoCyte Quanteon Flow Cytometer. The viable cell population was gated, and the cell cycle was analyzed using the built in analysis in the NovoExpress Software (Agilent).

### 5.8 Piezo1 Confocal Imaging

The ImmEdge Hydrophobic Barrier (PAP) Pen (Vector Laboratories) was used to draw hydrophobic barriers on the sterile Nunclon Delta Culture Slides (Thermo Scientific) prior to cell plating. The LNCaP and PC3 cells were lifted as described in the “Cell Culture” section. 7 × 10^4^ cells were plated within each hydrophobic barrier in 200 µL of complete media, and allowed to grow at 37°C and 5% CO_2_ for 48 h. Next, cells were fixed in a 4% PFA aqueous solution in HBSS++ (v/v) and incubated for 15 min. The cells were washed twice in HBSS‐/‐ for 5 min each, followed by permeabilization using Triton X‐100 (Sigma‐Aldrich) diluted to 1X in HBSS++ (v/v) at RT for 10 min. Samples were blocked in 5% BSA and 5% goat serum (diluted from 10% Normal Goat Serum (Life Technologies, Carlsbad, CA) (v/v) for 45 min at RT. Cells were incubated in the Piezo1 (extracellular domain) Polyclonal antibody (15939‐1‐AP, Proteintech, Rosemont, IL, USA) diluted at 1:100 (v/v) in 1% BSA for 1 h. Samples were stained with Goat anti‐Rabbit IgG (H+L) Cross‐Adsorbed Secondary Antibody, Alexa Fluor 488 (A11008, Invitrogen), 1 µg/mL DAPI nuclear stain (BD Pharmingen) and ActinRed 555 ReadyProbes Reagent (Invitrogen) for 30 min in the absence of light. Washing steps were performed between each step using HBSS‐/‐ for 5 min and all incubation steps were completed at RT. A drop of VECTASHIELD Antifade Mounting Medium (H‐1000, Vector Laboratories) was added to each sample, followed by coverslips and a few drops of nail polish for sealing. Slides were imaged on an LSM 900 Zeiss confocal microscope equipped with a 40X water immersion objective.

FIJI image software was used to measure the percentage of Piezo1 expression within each cell, using an in‐house developed macro [36]. The corrected total cell fluorescence (CTCF) was calculated using the following equation:

(4) $$\begin{array}{ccc} \mathrm{CTCF}\; & = & \;\mathrm{Integrated}\;\mathrm{Density}\\ & & -\left(A_{\mathrm{selected}\;\mathrm{cell}}\times \mathrm{Mean}\;\mathrm{fluorescenc}e_{\mathrm{background}}\right) \end{array}$$

where “*A*” is the area of the selected cell.

### 5.9 RNA Extraction

Prior to RNA extraction, 5 × 10^5^ cells were plated in a 6‐well plate overnight and maintained at 37°C and 5% CO_2_. The next day, RNA was extracted using the RNeasy kit (74104, QIAGEN) following the manufacturer's instructions. Briefly, the cells were washed two times in 1X HBSS‐/‐, and removed from the surface using a cell scraper and freshly prepared lysis buffer from the RNeasy kit. DNA digestion was performed using the RNase‐Free DNase Set (79254, QIAGEN). Following extraction, RNA purity was verified using a UV5Nano Spectrophotometer (Mettler Toledo).

### 5.10 Quantitative PCR

RNA was extracted as described in the “RNA Extraction” section above. Complementary DNA (cDNA) was synthesized using SuperScript VILO cDNA Synthesis Kit (11754050, Invitrogen) using the T100 Thermal Cycler (BIO‐RAD). cDNA expression was analyzed using real‐time PCR with the TaqMan Fast Advanced Master Mix for qPCR (4444963, Applied Biosystems), and fluorescence was detected in the FAM‐MGB channel according to the manufacturer's instructions. Samples were processed on the CFX96 Touch Real‐Time PCR System C1000 Touch Thermal Cycler (BIO‐RAD) using the following protocol: heat to 50°C for 2 min, 1 cycle for 20 s at 95°C, 40 cycles of 95°C for 3 s followed by 60°C for 30 s. Cycle threshold (Ct) values were calculated using CFX Maestro Software (Bio‐Rad). *GAPDH* was used as the housekeeping gene and used as an internal control for cDNA quantification and normalization of the amplified targets. *GAPDH* and *CALB2* primers were acquired from Thermo Fisher Scientific and their primer sequences are proprietary.

### 5.11 RNA‐Sequencing

Bulk RNA‐sequencing was performed by the VANTAGE core at Vanderbilt University Medical Center using RNA samples extracted as described in the “RNA Extraction” section. Sequencing was performed using the Illumina NovaSeq 6000 targeting (Paired‐End 150 bp) with an average of 50 × 10^6^ reads/sample. FASTQ files containing the PF reads were retrieved. We aligned the FASTQ sequences using Kallisto, and data were processed with the Bioconductor package DESeq2 using an R kernel in Jupyter Notebook [51]. Gene sets with a count across all samples ≤ 10 were filtered out. Heatmap plots were created in the Jupyter notebook. For the volcano plots, gene sets were manually filtered; significant genes are denoted as those with a ‐log_10_(P_adj_) > 2. Upregulated genes are those with a log2fold change (L2FC) > 2, and downregulated genes are those with a L2FC < ‐2. Volcano plots were replotted in Graphpad Prism.

### 5.12 Kaplan‐Meier Survival Analysis

Survival analyses were performed in R (v4.5.1). Gene‐level RNA‐seq counts and clinical annotations for The Cancer Genome Atlas Prostate Adenocarcinoma cohort (TCGA‐PRAD) were retrieved from the NCI Genomic Data Commons via TCGAbiolinks (workflow: STAR–Counts) [61], the SU2C/PCF Dream Team metastatic prostate cancer cohort [62], and the Taylor/MSKCC prostate cancer cohort (GSE21034) [63]. Raw counts were normalized to counts‐per‐million (CPM), then log‐transformed after adding a pseudo‐count of one for variance stabilization and visualization. The primary endpoint was overall survival (OS), defined as days to death; patients alive at last follow‐up were censored at that time. Patients were stratified into high vs low expression by the cohort median. Survival curves were estimated with the Kaplan–Meier method and compared using the log‐rank test. Hazard ratios (HRs) with 95% confidence intervals (CIs) were obtained from univariable Cox proportional‐hazards models; proportional‐hazards assumptions were evaluated using Schoenfeld residuals. Two‐sided p‐values are reported unless otherwise specified. Significance for the violin plots of *CALB2* expression across Gleason groups (≤ 6, 7, ≥ 8) was determined using the Kruskal–Wallis test. *CALB2*‐high (top) and *CALB2*‐low (bottom) outliers were defined as the top and bottom 5% of *CALB2* expression, respectively, within the data set.

### 5.13 PC3 mCherry‐Luciferase Transduction

To transduce the PC3 cells with mCherry‐luciferase, 5 × 10^4^ PC3 cells were plated in a 12‐well plate on day 1 and incubated at 37°C for 24 h prior to viral infection in complete media without PenStrep. On day 2, 8 µg/mL of Polybrene was added along with fresh complete media (no PenStrep) to each well undergoing transduction. For the transductions, cells were treated with 15 µL of HLUC‐Lv206 Firefly Luciferase+mCherry lentiviral particles (Genecopoeia) and incubated for 48 h at 37°C. On day 4, the media was removed, and 1 mL of fresh complete media was added. The cells were incubated overnight at 37°C. On day 5, the transduced samples were lifted using 0.25% trypsin‐EDTA and moved to a 6‐well plate. The cells were allowed to grow to confluence from there. A few weeks later, the cells were sorted for positive mCherry expression using the BD FACSAria III cell sorter at the Vanderbilt Flow Cytometry Shared Resource core. mCherry expression was validated using the NovoCyte Quanteon Flow Cytometer before cells were used in the orthotopic prostate cancer study.

### 5.14 Generation of CALB2 Knockout Cell Lines

*CRISPR Plasmid Design*: For *CALB2*, single guide RNA (sgRNA) target sites were determined using Benchling's built‐in CRISPR tool [91], selecting sgRNAs that would maximize on‐target and minimize off‐target cutting. For *CALB2*, our sgRNA is 5’–CTGTGCTTCAGGCAGCACGT–3’ with PAM 5’–GGG–3’, which is in Exon 4.

After sgRNA target selection, these were cloned into the lentiCRISPRv2‐mCherry backbone, following a published protocol [92]. LentiCRISPRv2‐mCherry was a gift from Agata Smogorzewska (Addgene plasmid # 99154; http://n2t.net/addgene:99154; RRID:Addgene_99154). These plasmids underwent traditional cloning workflow including bacterial transformation into electrocompetent Stbl3, recovery and growth/antibiotic selection at 30°C, and plasmid purification. Whole plasmid sequencing was used to verify plasmid sequence integrity.

#### 5.14.1 Lentiviral Production

24 h before transfection, 3.8 × 10^6^ HEK293T producer cells were plated on a 10 cm dish with antibiotic‐free DMEM (Gibco). Co‐transfection of psPAX2 packaging plasmid, pMD2.G envelope plasmid, and each lentiCRISPRv2‐mCherry transfer plasmid was performed using jetPRIME reagent (Polyplus), following manufacturer's instructions. psPAX2 was a gift from Didier Trono (Addgene plasmid # 12260; http://n2t.net/addgene:12260; RRID:Addgene_12260); pMD2.G was a gift from Didier Trono (Addgene plasmid # 12259; http://n2t.net/addgene:12259; RRID:Addgene_12259). 24 and 48 h after transfection, each virus prep was collected, filtered using 0.45 µm PVDF filter, concentrated 10X using Lenti‐X Concentrator (Takara Bio, Cat. No. 631231), and snap‐frozen for cryovial storage at −80°C. 72 h post‐transfection, producer cells were run on a NovoCyte Quanteon Flow Cytometer to confirm viable lentiviral particle production, as indicated by positive mCherry expression.

#### 5.14.2 Lentiviral Transduction of Target Cells for CRISPR Knockout and Sorting

Prior to transductions, 7.5 × 10^4^ LNCaP and PC3 cells were plated in a 12‐well dish in PenStrep‐free complete media and allowed to grow overnight. 24 h later, the cells were treated with lentiviral particles and 8 µg/mL of polybrene (CAS 28728‐55‐4, Santa Cruz Biotechnology, Dallas, TX, USA). The PC3 cells were treated with the *CALB2*‐targeting lentiviral particles. The cells were transduced for 48 h. After, the cells were moved to a T75 flask and allowed to grow to confluency to be sorted for positive mCherry expression using the FACS SONY MA900 sorter managed by the Shared Equipment Authority at Rice University.

#### 5.14.3 Target Cell Genotyping for Knockout Validation

gDNA of each sample group was extracted from 5 × 10^5^ cells using the DNeasy Blood & Tissue Kit (69504, QIAGEN), following manufacturer's protocol and eluted in nuclease‐free water. Then, PCR amplification of the ∼500 bp genomic region surrounding the sgRNA target site was performed as instructed by the manufacturer with Q5 High‐Fidelity DNA Polymerase (M0491S, New England Biolabs) and custom PCR primers, designed with Benchling's built‐in primer tool [93]. Gel electrophoresis was performed with 1.5% Agarose Gel, and correct bands were gel extracted using the GeneJET Gel Extraction Kit (K0691, Thermo Scientific), following manufacturer's instructions and eluted in nuclease‐free water. Lastly, these purified PCR products were Sanger Sequenced and analyzed using EditCo's Inference of CRISPR Edits (ICE) online software (https://ice.editco.bio) or Netherlands Cancer Institute's Tracking of Indels by Decomposition (TIDE) online software (https://tide.nki.nl) [94].

### 5.15 Subcutaneous In Vivo Study

Male eight‐week‐old nude (24102262‐NU/NU) mice were obtained from Charles River Laboratory (Wilmington, MA, USA). This study was approved by the Institutional Review Board protocol #M1700009‐02. All mice received identical care and were maintained in sterile housing conditions. Mice were monitored by veterinary staff in the Vanderbilt University Division of Animal Care (DAC).

LNCaP and PC3 parental and MR cells were lifted and counted with a hemocytometer as described in the “Cell Culture” section. Cells were washed twice with HBSS‐/‐ at 300 x g for 5 min. 1×10^6^ PC3 cells were resuspended in a 250 µL mixture of 1:1 (v/v) PBS‐/‐ and Matrigel (354248, Corning Life Sciences, Tewksbury, MA, USA). While anesthetized with 3%–5% isoflurane, both flanks on the mice were inoculated with 1 × 10^6^ cells using 29 G Exel insulin needle and syringe (EUO52913, Air‐Tite). Tumors were measured using calipers 2–3 times per week while mice were anesthetized. Tumor volume was measured using the following, where “L” is length and “W” is tumor width:

(5) $$\mathrm{Tumor}\;\mathrm{volume}\;=\frac{L\cdot W^{2}}{2}$$

At endpoint, tumors were resected and fixed in 10% neutral buffered formalin (NBF) supplied by the Vanderbilt Translational Pathology Shared Resource (TPSR) for 48 h.

### 5.16 Orthotopic Prostate Cancer Study

Six to eight‐week‐old male NOD/SCID mice (Strain Code 394) were purchased from Charles River Laboratory (Wilmington, MA, USA). This study was approved by Institutional Animal Care and Use Committee at Rice University, protocol #IACUC‐24‐112‐RU. All mice received identical care and were maintained in sterile housing conditions. Mice were monitored daily by the veterinary staff within the Animal Research Facility at Rice University.

The cells were prepped for surgery as described in the “Cell Culture” section, with one extra wash in 1X PBS‐/‐ after lifting. Approximately 1 h before surgery, mice were administered 1.3 mg/mL of Ethiqa XR Buprofenor (CIII) VI (07‐894‐7617, Patterson Veterinary, Greeley, CO, USA) via subcutaneous injection. Mice were anesthetized with 3%–5% isoflurane in an induction chamber and placed in supine position, remaining under anesthesia with a nose cone, while resting on a heating pad. Mice remained in sterile condition during the surgery. Nair (Veet, Reckitt Benckiser, Parsippany, NJ, USA) was used to remove the fur from the injection site, and the skin area was disinfected with povidone‐iodine prep pads 10% w/v (Dynarex Corporation, Israel) followed by alcohol pre pads (Dealmed, Brooklyn, NY, USA), three times.

A small incision was made on the lower abdomen using scissors, taking care to avoid the bladder. Sterile cotton swabs (McKesson Richmond, VA, USA) were used to expose the seminal vesicle and anterior lobe in preparation for the injection. The 29 G Exel insulin needle and syringe was used to slowly inject 1 × 10^6^ PC3 cells resuspended in 50 µL of Matrigel diluted in half with 1X PBS‐/‐. The needle was slowly removed following injection, and a sterile cotton swab was held over the injection site for 1–2 s to prevent leaking. The seminal vesicle was placed back into the abdominal cavity. The abdominal wall was closed using ETHICON Coated VICRYL absorbable sutures size 5/0 (07‐807‐0542, Patterson Veterinary) and the skin layer was closed using 3M Vetbond Tissue Adhesive (07‐805‐5031, Patterson Veterinary). Mice were placed back into a sterile, clean cage on a heating pad following surgery and were monitored until they woke up. Tumors were monitored via bioluminescence imaging (BLI) twice per week using the In Vivo Imaging System (IVIS) (Perkin Elmer) managed by the Shared Resource Facility (SEA) at Rice University. 28 mg/kg Pierce D‐Luciferin, Monopotassium Salt (88294, Thermo Scientific) was injected in the intraperitoneal (IP) cavity prior to imaging. Then, mice were placed under 3%–5% isoflurane prior to and during imaging. BLI images were analyzed using the Living Image Software Version 4.8.2 (Revvity Inc., Waltham, MA, USA; RRID:SCR_014247) with the same min and max used for all timepoints and mice for the course of the study. For weeks 3–7 of BLI, the D‐Luciferin concentration was 14 mg/kg. The total flux readouts for these weeks were corrected according to normalization factors determined based on the weeks when 28 mg/kg was used. At day 90 post‐op, the mice were euthanized. Cardiac puncture was performed to isolate bloodborne CTCs, and tumors and organs were removed following perfusion with 0.9% normal saline (ViP Care) and 10% NBF (TissuePro). The Center for Comparative Medicine at Baylor College of Medicine routinely processed, paraffin embedded and sectioned the tumors and tissues at 5 µm. Primary tumor volume was measured ex vivo using calipers and calculated using Equation 5.

### 5.17 Immunohistochemistry Staining

The Center for Comparative Medicine at Baylor performed hematoxylin and eosin (H&E) staining for each of the tissue sections submitted. Samples were submitted to iHisto for slide scanning.

### 5.18 Immunofluorescence Mouse Tissue Staining

5 µm, unstained tissue sections of the primary tumors were retrieved from the Center for Comparative Medicine at Baylor. Slides were placed in a slide holder and fully submerged in Epredia Signature Series Clear‐Rite 3 (Fisher Scientific), followed by 3 sequential 3 min incubations in fresh solution, with the slide rack briefly wiped between transfers to minimize carryover. Slides were then rehydrated through graded ethanols (diluted in MQ water), consisting of 3 sequential 2 min incubations in 100% ethanol (each in separate containers), followed by a single two min incubation in 95% and 70% ethanol. After rehydration, slides were immersed in distilled water for 5 min.

For antigen retrieval, slides were placed into a heat‐resistant glass slide holder. Tris‐EDTA + 0.05% Tween‐20 solution at a pH of 9.0. Tris base (Fisher Scientific) and Ethylenediaminetetraacetic acid (EDTA) disodium salt dihydrate (Millipore Sigma) were used to prep this solution. Boiling Tris‐EDTA was added directly to the slide rack, and the rack was placed into a pre‐warmed rice cooker for 30 min. Slides were then cooled for 30 min at RT. Slides were washed 3x in MQ water for 5 min each.

Tissue samples were outlined with the ImmEdge Hydrophobic Barrier (PAP) pen and were blocked in 5% BSA and 5% goat serum for 1 h at RT. Next, calretinin (E7R60) XP Rabbit mAB primary antibody, diluted at 1:100 (Cell Signaling Technology) in 5% BSA and 5% goat serum was incubated overnight at 4°C, with a small piece of parafilm on top. After incubation, the samples were washed 3x for 10 min in 0.04% Tween in 1X PBS. Samples were stained with Goat anti‐Rabbit IgG (H+L) Cross‐Adsorbed Secondary Antibody, Alexa Fluor 488 (A11008, Invitrogen) diluted 1:250 and 1 µg/mL DAPI nuclear stain (BD Pharmingen) diluted in 5% BSA and 5% goat serum, incubated for 1 h at RT in the dark. Following incubation, slides were washed 3x for 10 min in 0.04% Tween in 1X PBS. 2–3 drops of VECTASHIELD PLUS Antifade Mounting Medium (H‐1900‐2, Vector Laboratories) mounting media was added to each tissue section and slides were coverslipped and sealed with nail polish. Tissue samples were imaged using an LSM 900 Zeiss confocal microscope equipped with a 63X oil immersion objective.

### 5.19 Immunofluorescence Human Tissue Microarray Staining

The following human tissue microarrays were used; Human Prostate Tissue MicroArray (Cancer) from Novus Biologicals (NBP2‐30169) and PR821 from TissueArray.com. The microarrays were first dried at 60°C in an oven (Model 664, Fisher Scientific). Slides were placed in a slide holder and fully submerged in Epredia Signature Series Clear‐Rite 3 (Fisher Scientific), followed by 4 sequential 5 min incubations in fresh solution, with the slide rack briefly wiped between transfers to minimize carryover. Slides were then rehydrated through graded ethanols (diluted in MQ water), consisting of 2 sequential 3 min incubations in 100% ethanol (each in separate containers), followed by 2 sequential 3 min washes in 95% and 75% ethanol. After rehydration, slides were immersed in distilled water for 5 min.

Antigen retrieval was completed the same way as described in the “Immunofluorescence Mouse Tissue Staining” section above. Briefly, microarray slides were placed into a slide rack containing boiling Tris‐EDTA + 0.05% Tween‐20 solution, and then placed into a rice cooker with boiling water for 30 min, then allowed to cool for 30 min. Slides were washed 3x in MQ water for 5 min each.

Tissue samples were outlined with the ImmEdge Hydrophobic Barrier (PAP) pen and were blocked in 5% BSA and 5% goat serum for 1 h at RT. Next, calretinin (E7R60) XP Rabbit mAB primary antibody, diluted at 1:100 (Cell Signaling Technology) in 5% BSA and 5% goat serum was incubated overnight at 4°C, with a small piece of parafilm on top. After incubation, the samples were washed 3x for 10 min in 0.04% Tween in 1X PBS. Samples were stained with Invitrogen Goat anti‐Rabbit IgG (H+L) Cross‐Adsorbed Secondary Antibody, Alexa Fluor 647 (A21244, Invitrogen) diluted 1:250, Alexa Fluor 488 anti‐human PSMA (FOLH1) Antibody (342506, BioLegend) diluted 1:100, and 1 µg/mL DAPI nuclear stain (BD Pharmingen) diluted in 5% BSA and 5% goat serum, incubated for 1 h at RT in the dark. Following incubation, slides were washed 3x for 10 min in 0.04% Tween in 1X PBS. 2–3 drops of VECTASHIELD PLUS Antifade Mounting Medium (H‐1900‐2, Vector Laboratories) mounting media was added to each tissue section and slides were coverslipped and sealed with nail polish. Tissue samples were imaged using an LSM 900 Zeiss confocal microscope equipped with a 20X objective. Tissue samples were imaged by tiling at the center point of each section.

### 5.20 Immunofluorescence Mouse and Human Tissue Analysis

Calretinin CTCF was quantified using ImageJ to process the images [36]. Briefly, a grid was applied to the composite images, to allow for random selection of either five (mouse) or ten (human) sections of the tissue samples to be selected for quantification. Integrated density and area were measured. Background sections were then selected to assess mean background intensity, so that the CTCF could be calculated using Equation 4.

### 5.21 Statistical Analysis

Data are reported as mean and standard error of the mean (SEM). Experiments included at least three independent replicates. Statistical significance is indicated as: *p < 0.05, **p < 0.01, ***p < 0.005, and ****p < 0.001 for significance; otherwise, no significant difference was found. GraphPad Prism software was used to perform statistical analyses and produce figures for this article.

### 5.22 Ethics Approval

The protocol for the subcutaneous in vivo mouse study (Figure S7) in this article was approved by the Vanderbilt IACUC protocol #M1700009‐02 and mice were monitored by staff from the Division of Animal Care at Vanderbilt University. The protocol for the orthotopic PCa in vivo mouse study (Figure 7) was approved by the Rice University protocol #IACUC‐24‐112‐RU and mice were monitored by the staff from the Animal Research Facility at Rice University.

## Author Contributions

Conceptualization: A.R.F., M.R.K., Methodology: A.R.F., A.C.L., P.T., J.A.D., M.S.C., S.J.R., E.A., S.V.K., and M.R.K., Investigation: A.R.F., A.C.L., P.T., M.S.C., S.J.R., E.A., A.C., S.V.K., and M.R.K., Animal studies: A.R.F., A.C.L., J.A.D., S.J.R., and S.V.K., Data curation: A.R.F., P.T., and E.A., Visualization: A.R.F., Formal analysis: A.R.F., E.A., Funding acquisition: A.R.F. and M.R.K., Project administration: M.R.K., Supervision: C.R.K. and M.R.K., Writing – original draft: A.R.F. and M.R.K., Writing – review & editing: A.R.F. and M.R.K.

## Funding

This work was supported by the United States National Institute of Health grant number R01CA256054 (MRK), Cancer Prevention and Research Institute of Texas (CPRIT) Grant No. RR230029 (MRK), National Science Foundation Graduate Research Fellowship (ARF), grant number 2021314768, Genetic Design and Engineering Center (GDEC) at Rice University, which is funded by CPRIT RP210116. The Vanderbilt Flow Cytometry Shared Resource is supported by the Vanderbilt Ingram Cancer Center (P30 CA068485) and the Vanderbilt Digestive Disease Research Center (DK058404). This work was done in part using resources of the Shared Equipment Authority at Rice University. FACS sorting at Vanderbilt University was completed in the Vanderbilt Flow Cytometry Shared Resource using the BD FACSAria III Cell Sorter.

## Supporting information




**Supporting File**: advs76535‐sup‐0001‐SuppMat.pdf.

## Data Availability

All data except for the RNA‐sequencing data is available in the main text or the supplementary materials. The data discussed in this publication have been deposited in the NCBI's Gene Expression Omnibus and are accessible through GEO Series accession number GSE337264 (https://www.ncbi.nlm.nih.gov/geo/query/acc.cgi?acc=GSE337264) [95, 96].
