# Supplementary material for: CALB2 is a Mechanoresistance Gene in Metastatic Prostate Cancer
Source: Adv Sci (Weinh). 2026 Jul 20;13(55):e76535. doi: 10.1002/advs.76535 (PMC13383692; doi:10.1002/advs.76535)
Supplement: Supplementary file 1 [file ADVS-13-e76535-s001.pdf]

Supporting Information

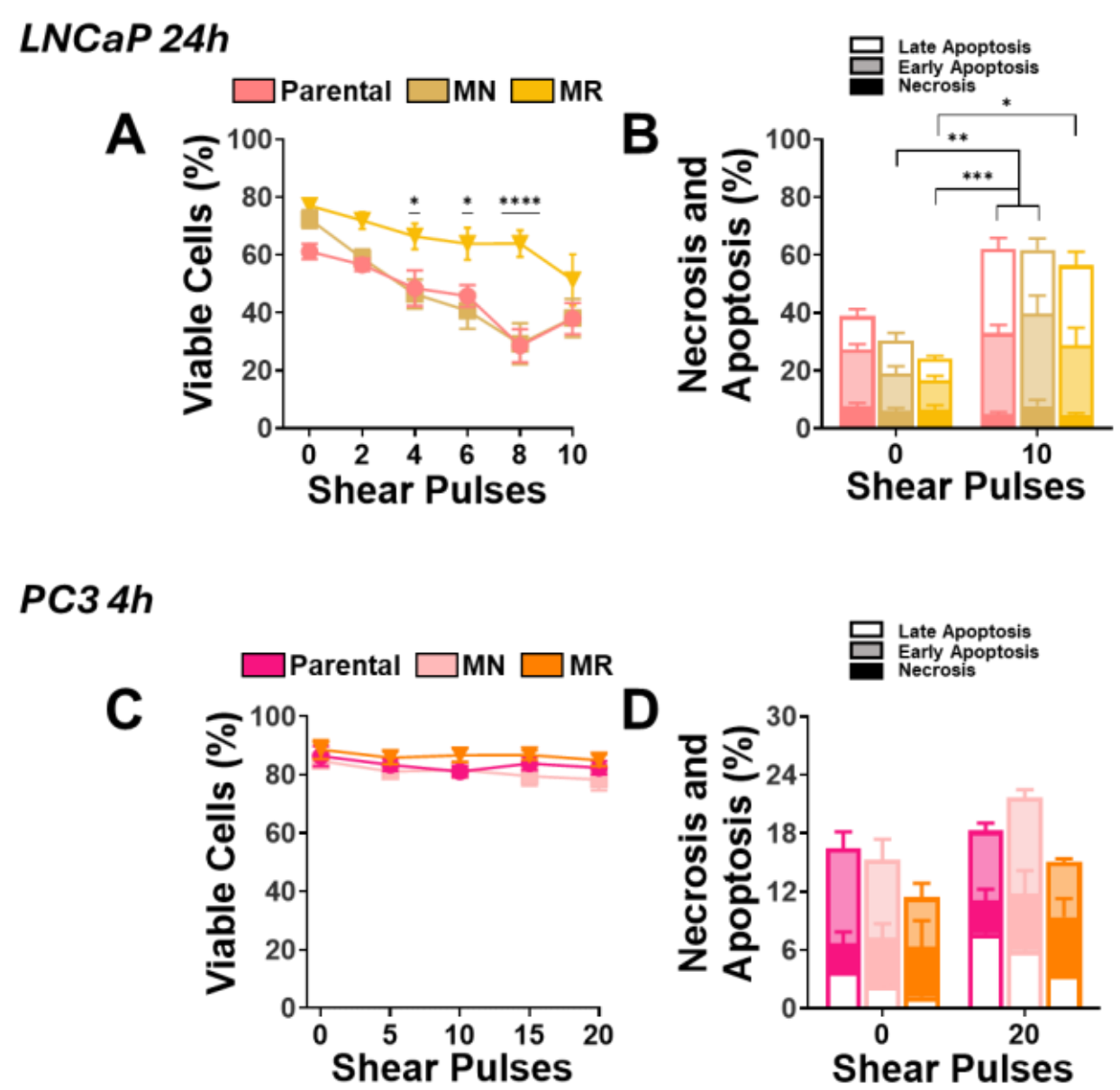

Figure S1. Viability following high-intensity fluid shear stress (HI FSS) exposure. Percentage of (**A**) viable and (**B**) total apoptosis and necrosis in the LNCaP cells 24 h following FSS exposure. Percentage of (**C**) viable and (**D**) total apoptosis and necrosis in the PC3 cells 4 h following FSS exposure. *n* = 3 – 6 independent experiments. (A – D) Two-way ANOVA. *p<0.05, **p<0.01, ***p<0.005, ****p<0.0001. Error bars represent mean ± SEM.

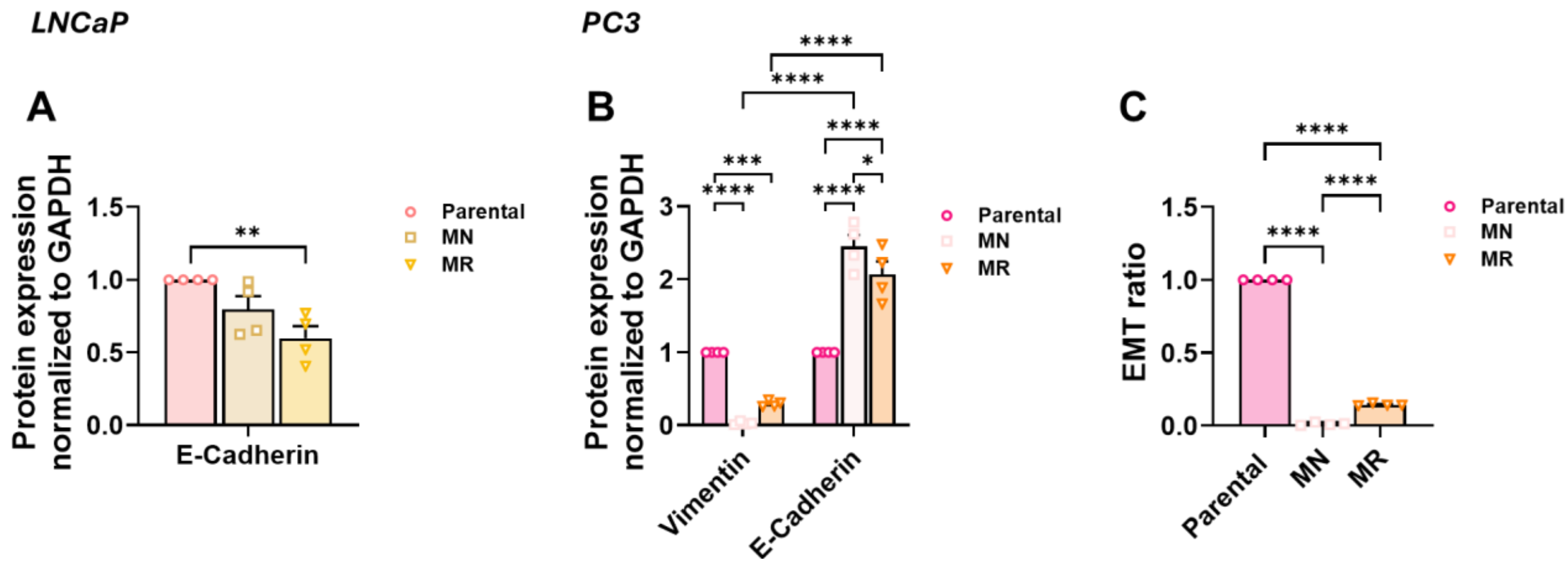

Figure S2. Epithelial-to-mesenchymal transition (EMT) protein expression. (**A**) E-cadherin protein expression in LNCaP cells. (**B**) Vimentin and E-cadherin protein expression in the PC3 cells. (**C**) EMT ratio in PC3 cells (normalized vimentin expression/normalized E-cadherin expression. Protein expression is normalized to GAPDH and then to the parental condition. $n = 3 – 4$ independent experiments. (A,C) One-way ANOVA. (B) Two-way ANOVA. $^*p<0.05$, $^{**}p<0.01$, $^{***}p<0.005$, $^{****}p<0.0001$. Error bars represent mean ± SEM.

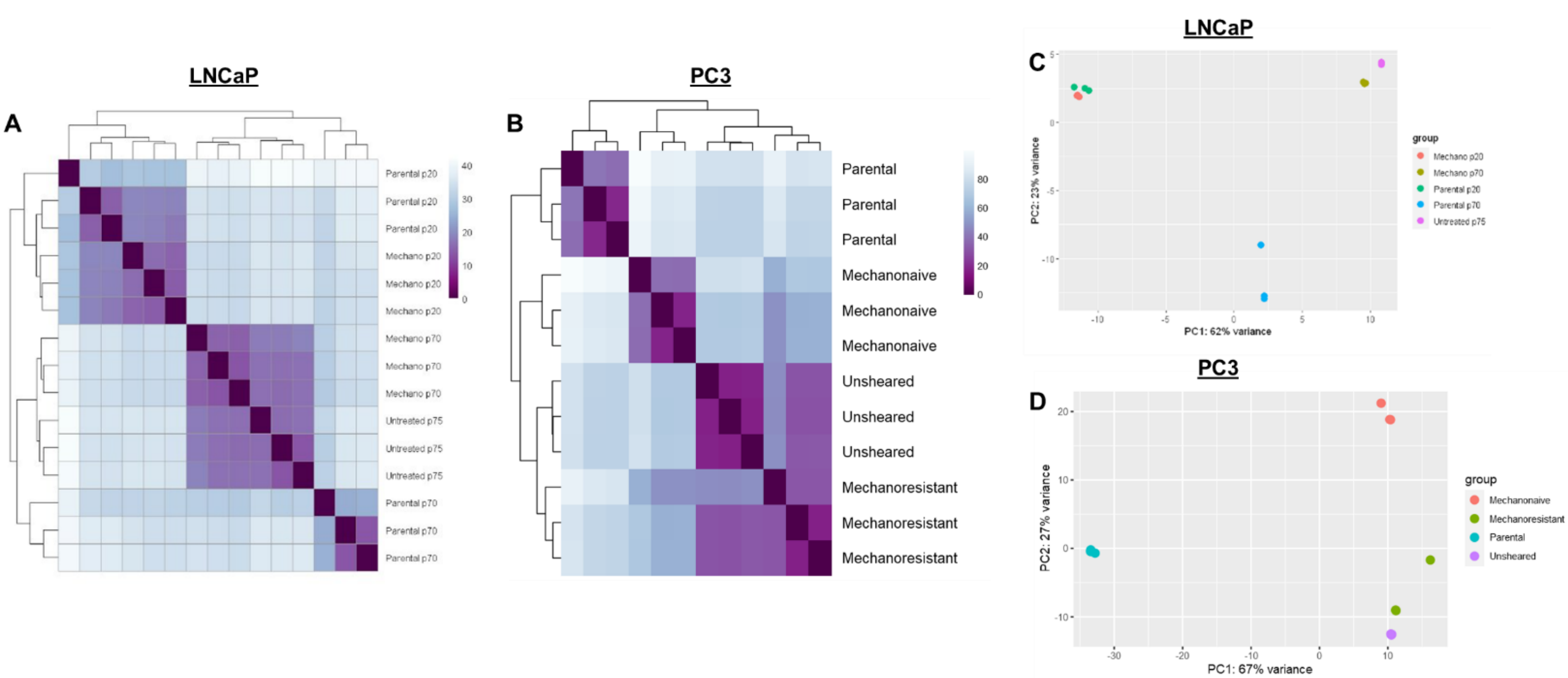

Figure S3. RNA-sequencing sample distance plots. (**A**) Euclidean distance plot for the LNCaP cells. (**B**) Principal component analysis (PCA) plot for the LNCaP cells. (**C**) Euclidean distance plot for the PC3 cells. (**D**) PCA plot for the PC3 cells. All plots generated using DESeq2 package in R-Jupyter Notebook.

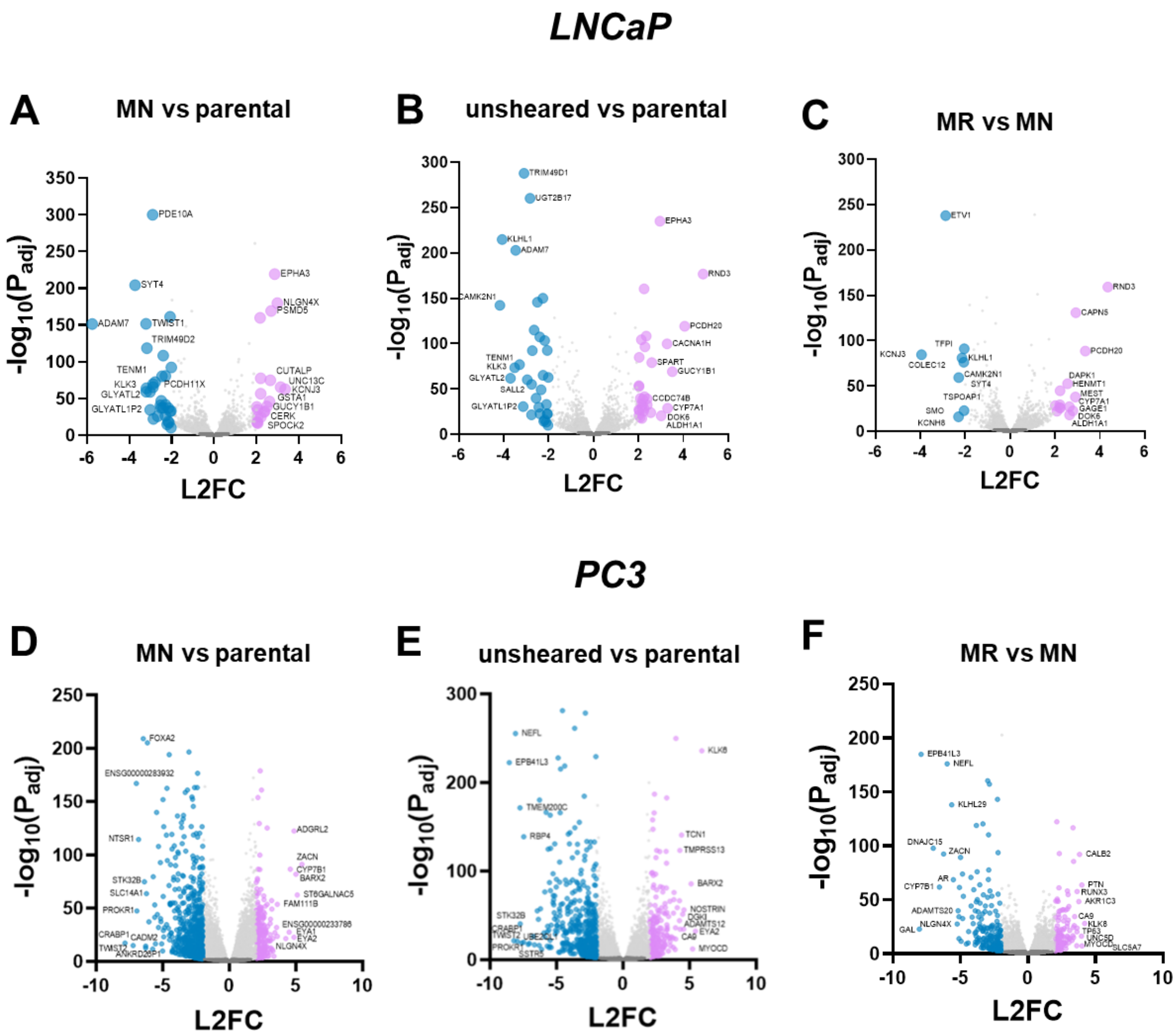

Figure S4. Volcano plot analysis following RNA-sequencing. Volcano plots showing the significantly up- and down-regulated genes in the: (**A**) mechanonaïve (MN) vs parental cells, (**B**) unsheared mechanoresistant (MR) vs parental cells and (**C**) MR vs MN LNCaP cells. Volcano plots showing the significantly up- and down-regulated genes in the: (**D**) MN vs parental cells, (**E**) unsheared vs parental cells and (**F**) MR vs MN PC3 cells. Data analyzed using DESeq2 in the R-Jupyter Notebook and graphs created in Prism GraphPad. *n* = 3 RNA samples per condition.

Table S1. Significantly up- or downregulated genes expressed by the mechanoresistant (MR) cells. Genes specific to the MR cells that were up- and down-regulated in comparison to the parental and MN conditions taken from the volcano plot analysis conducted using DESeq2.

significantly Downregulated genes:	significantly Upregulated genes:
**LNCaP**	
• *KLHL1*<br>• *CAMK2N1*	• *RND3*<br>• *PCDH20*<br>• *CYP7A1*<br>• *ALDH1A1*<br>• *DOK6*
**PC3**	
• *EPB41L3*<br>• *NEFL*<br>• *C1QTNF5*	• *DGKI*<br>• *TCN1*<br>• *SLC5A7*<br>• *MYOCD*<br>• *CALB2*

Figure S5. ***CALB2*** **expression grouped by Gleason Scores.** *CALB2* expression levels in primary tumor samples (patient-level) grouped by Gleason Score using the (**A**) SU2C/PCF Dream Team Metastatic PCa (n = 185 patients) and (**B**) Taylor/MSKCC PCa (n = 201 patients) cohorts.

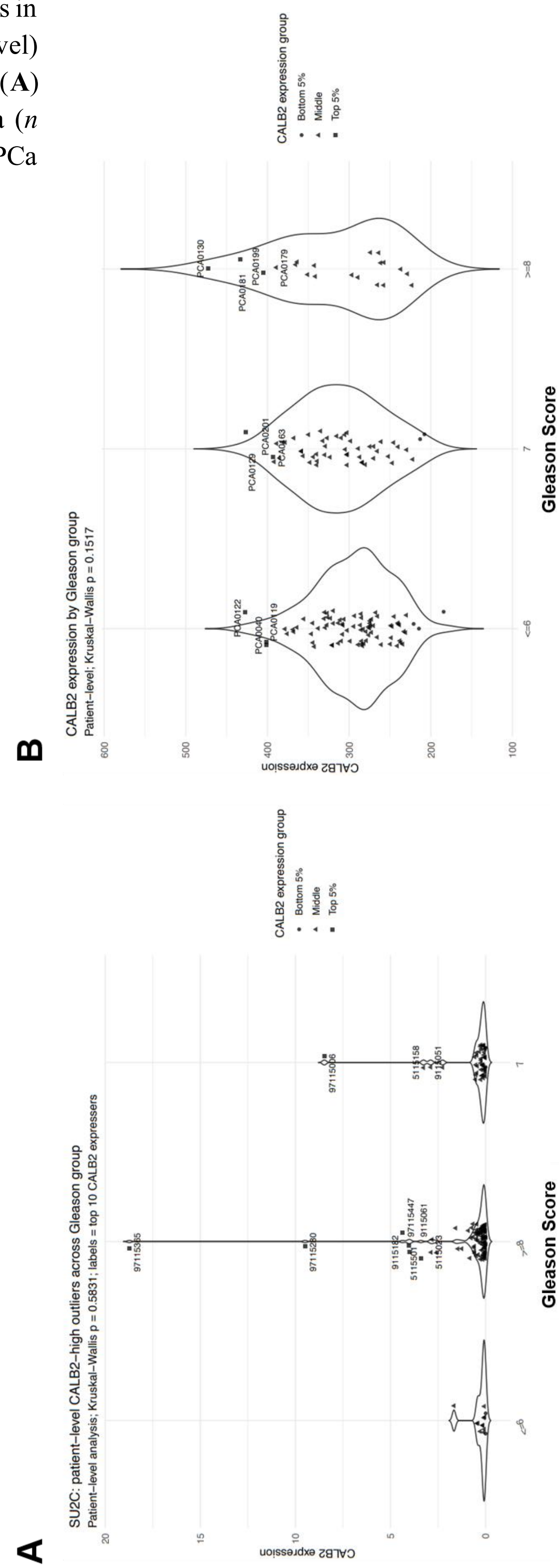

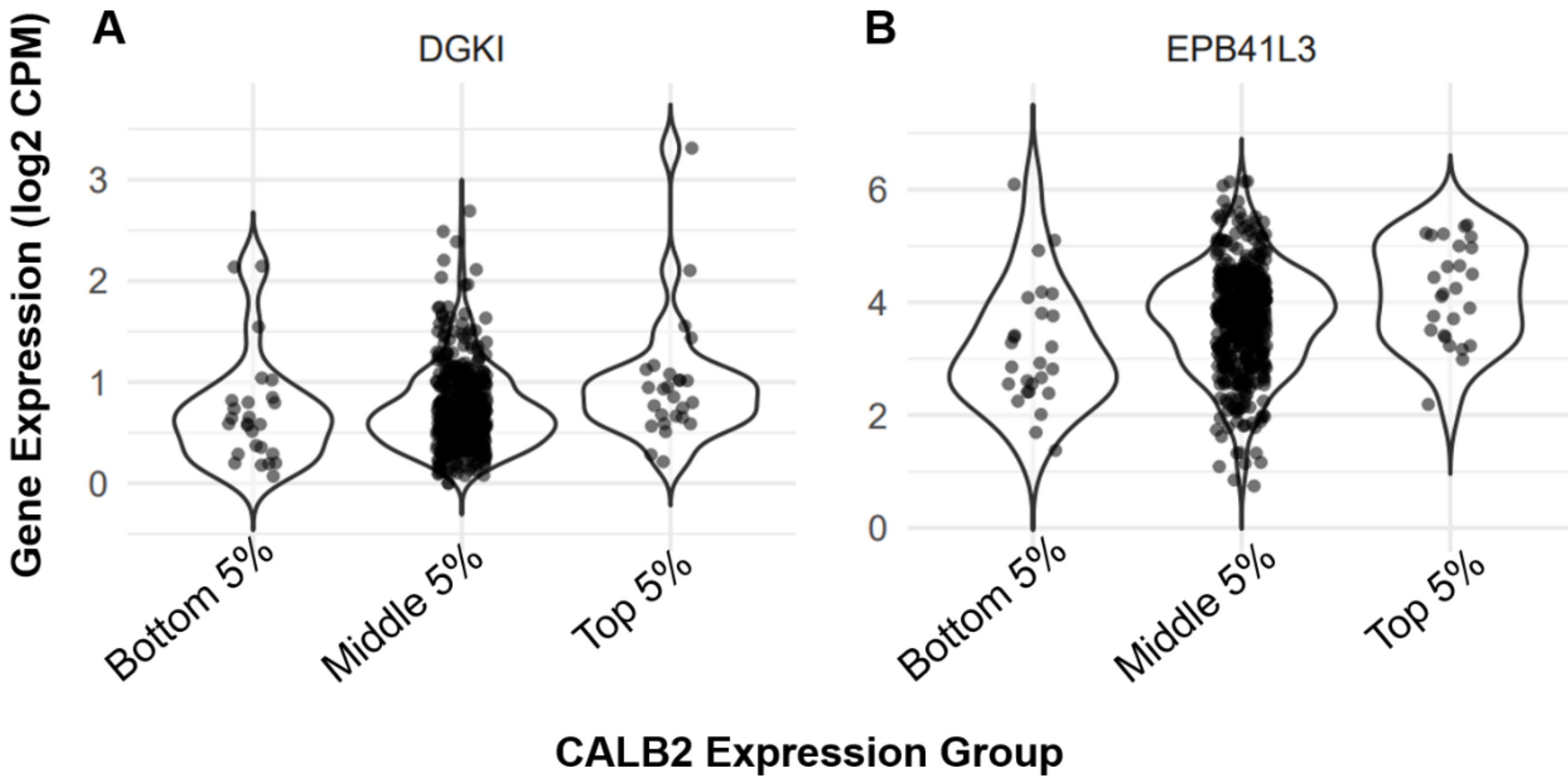

Figure S6. Association of *CALB2* expression in primary tumor samples with mechanoadaptive genes. *CALB2* mRNA expression from the TCGA-PRAD cohort, in which *CALB2*-high and *CALB2*-low outliers were defined as the top and bottom 5% of *CALB2* expression, respectively. Correlation with (A) *DGKI* and (B) *EPB41L3* (n = 501 primary tumor samples).

Table S2. Primary tumor sample–level associations between *CALB2* expression groups and candidate genes. Median expression values for the top 5%, middle, and bottom 5% of *CALB2* expressers are shown, along with Kruskal–Wallis tests across *CALB2* extreme groups and Spearman correlations with *CALB2*. Data from the TCGA-PRAD cohort (*n* = 501 primary tumor samples).

dataset	gene	median_top5	median_middle	median_bottom5	kruskal_p_across_CALB2_extreme_groups	kruskal_fdr	spearman_rho_with_CALB2	spearman_fdr
Primary tumor samples	EPB41L3	4.1998	3.7771	2.8884	0.0006	0.0062	0.3079	0.0000
Primary tumor samples	DGKI	0.8923	0.6551	0.5880	0.0225	0.1124	0.1818	0.0002
Primary tumor samples	MMP9	2.8802	2.7507	2.4470	0.1400	0.3882	0.0595	0.3062
Primary tumor samples	KLK3	13.9889	14.2610	13.9554	0.1553	0.3882	−0.0129	0.7786
Primary tumor samples	FOLH1	8.3698	8.8794	9.1528	0.2003	0.4006	−0.1034	0.0686
Primary tumor samples	CAMK2N1	6.0086	5.6183	5.4995	0.3991	0.6651	0.0819	0.1338
Primary tumor samples	NEFL	1.2090	0.7850	0.5507	0.5357	0.6697	0.0454	0.4431
Primary tumor samples	TCN1	0.2013	0.3030	0.3187	0.5103	0.6697	−0.0126	0.7786
Primary tumor samples	KLHL1	0.0309	0.0277	0.0512	0.9626	0.9946	−0.0371	0.5090
Primary tumor samples	CYP7A1	0.0421	0.0430	0.0463	0.9946	0.9946	0.0901	0.1097

Table S3. Primary tumor sample–level co-high enrichment analysis. Shown are the numbers of *CALB2*-high samples overlapping with high expression of each candidate gene, along with corresponding odds ratios and Fisher's exact test statistics. Data from the TCGA-PRAD cohort (*n* = 501 primary tumor samples).

dataset	gene	n_CALB2_top5	n_gene_high	n_CALB2_top5_and_gene_high	odds_ratio	fisher_p	fisher_fdr
Primary tumor samples	EPB41L3	26	26	3	2.5563	0.1456	0.4854
Primary tumor samples	DGKI	26	26	3	2.5563	0.1456	0.4854
Primary tumor samples	KLK3	26	26	3	2.5563	0.1456	0.4854
Primary tumor samples	CYP7A1	26	26	0	0.0000	0.3867	0.7735
Primary tumor samples	TCN1	26	26	0	0.0000	0.3867	0.7735
Primary tumor samples	KLHL1	26	26	1	0.7204	1.0000	1.0000
Primary tumor samples	CAMK2N1	26	26	1	0.7204	1.0000	1.0000
Primary tumor samples	NEFL	26	26	1	0.7204	1.0000	1.0000
Primary tumor samples	MMP9	26	26	1	0.7204	1.0000	1.0000
Primary tumor samples	FOLH1	26	26	1	0.7204	1.0000	1.0000

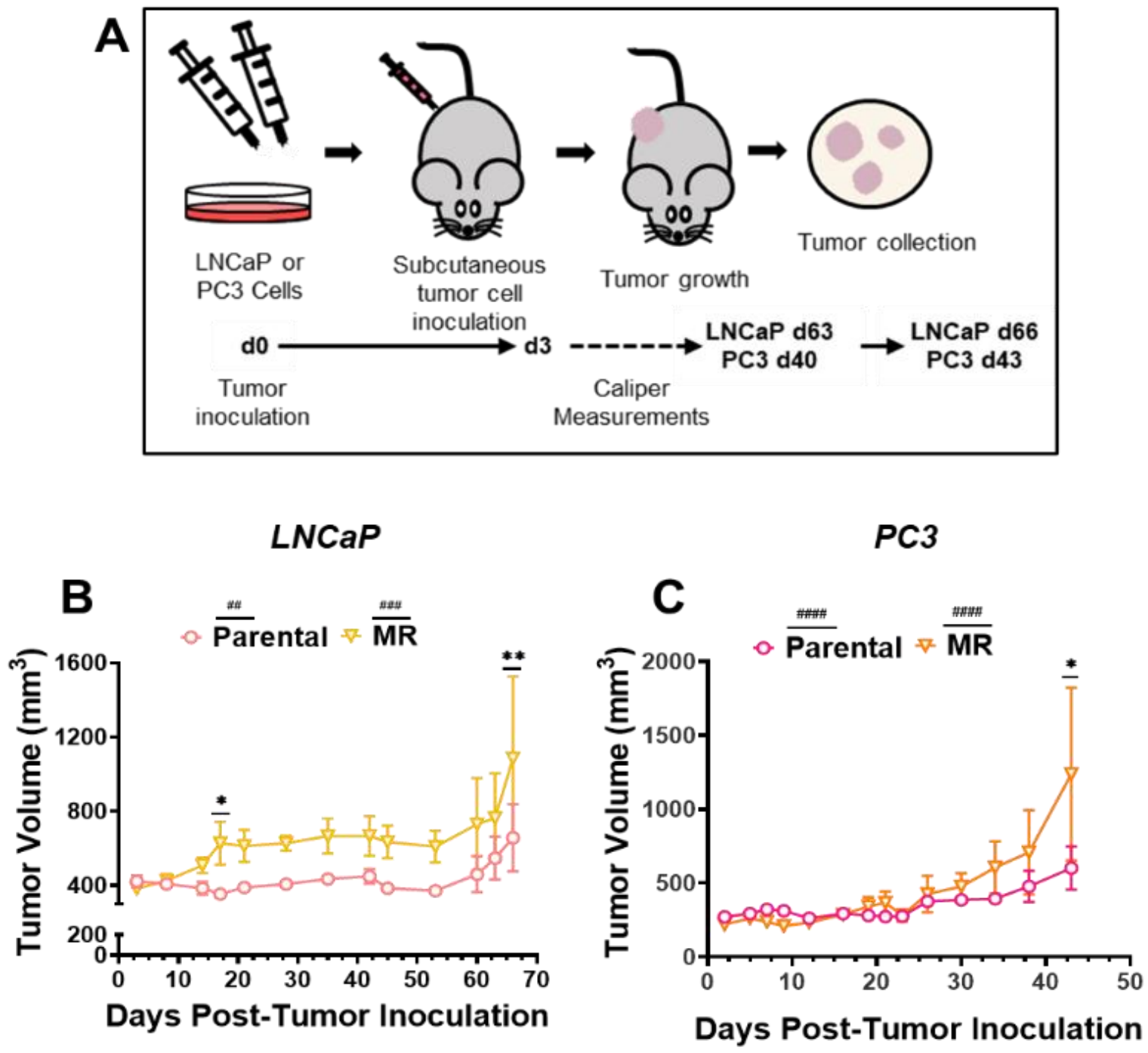

Figure S7. Tumorigenicity analysis of the mechanoresistant (MR) cells. (**A**) Timeline for the in vivo tumor model study. Average tumor volume for the (**B**) LNCaP and (**C**) PC3 cells measured using calipers (**Equation 5**). *n* = 6 mice per condition. (B,C) Two-way ANOVA and simple linear regression to confirm significant deviation from zero, ##<0.01, ###<0.005, ####p<0.0001. *p<0.05, **p<0.01, ***p<0.005, ****p<0.0001. Error bars represent mean ± SEM.
